# Chemical biology tools for probing transcytosis at the blood–brain barrier†
†Electronic supplementary information (ESI) available. See DOI: 10.1039/c9sc04024b


**DOI:** 10.1039/c9sc04024b

**Published:** 2019-10-08

**Authors:** Rhiannon Beard, David C. A. Gaboriau, Antony D. Gee, Edward W. Tate

**Affiliations:** a Department of Chemistry , Imperial College London , Wood Lane , London , W12 0BZ , UK . Email: rhiannon.beard@gmail.com ; Email: e.tate@imperial.ac.uk; b Facility for Imaging by Light Microscopy , Imperial College London , Exhibition Road , London SW7 2AZ , UK; c Division of Imaging Sciences , King's College London , St Thomas' Hospital , SE1 7EH , UK; d Francis Crick Institute , 1 Midland Road , London NW1 1AT , UK

## Abstract

Absorptive- and receptor-mediated transcytosis (AMT/RMT) are widely studied strategies to deliver therapeutics across the blood–brain barrier (BBB). However, an improved understanding of the mechanism surrounding trafficking is required that could promote delivery. Accordingly, we designed a flexible platform that merged AMT and RMT motifs on a single scaffold to probe various parameters (ligand, affinity, valency, position) in a screening campaign. During this process we adapted an *in vitro* BBB model to reliably rank transcytosis of the vehicle library. Our results demonstrate heightened uptake and trafficking for the shuttles, with a structure–activity relationship for transcytosis emerging. Notably, due to their small size, the majority of shuttles demonstrated increased permeation compared to transferrin, with the highest performing shuttle affording a 4.9-fold increase. Consequently, we have identified novel peptide conjugates that have the capacity to act as promising brain shuttles.

## Abstract 2

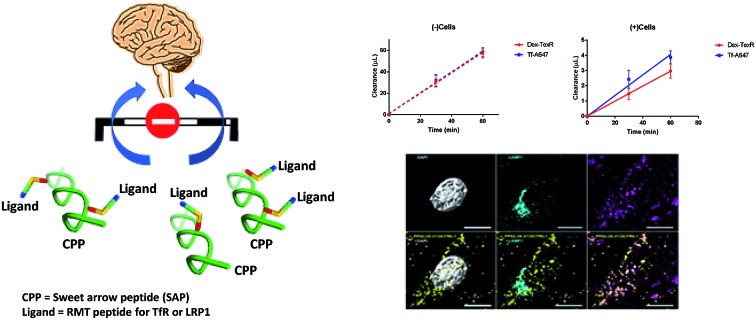
A BBB vehicle library is validated, establishing a SAR for uptake. Merging AMT/RMT motifs chemoselectively increased permeability.

## Introduction

A major hurdle hindering the diagnosis and treatment of neurological disorders is the difficulty for biotherapeutics to enter the central nervous system (CNS). This is due to the blood–brain barrier (BBB), which is comprised of tightly connected polarized endothelial cells that limit the passage of hydrophilic components and prevents the accumulation of material for transport at the BBB.^1^ Despite these limitations, specialized endogenous transport mechanisms exist to allow the transcytosis of nutrients and ions, thus enabling CNS homeostasis. Of these, absorptive mediated-(AMT) and receptor mediated-transcytosis (RMT) pathways are key vesicular based transport systems which have become long-standing approaches for drug delivery to the CNS.^2^ While these routes have become widely exploited by conjugating molecules restricted by the barrier to those which have this capacity, transcytosis at the BBB is more complex than initially thought, and delivery of therapeutics and biologicals remains modest.^3^,^4^

Whilst there is limited experimental data surrounding the molecular basis of uptake and trafficking for CNS delivery, it has been demonstrated that dissociation from receptors on the brain side is essential for trafficking mediated by the transferrin receptor (TfR), the quintessential receptor for RMT.^5^ Therefore transcytosis is more likely when the overall affinity towards the receptor is moderate to low,^5^ or when bivalent engagement of the receptor is discouraged to limit avidity.^6^

**Fig. 1:**
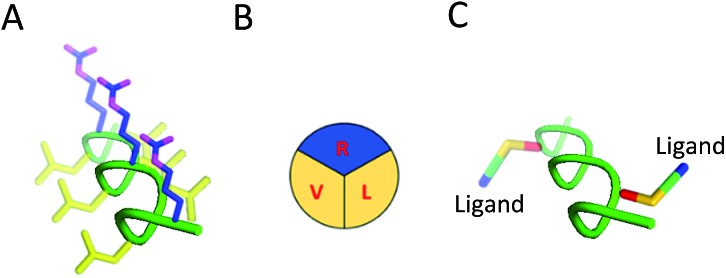
Structure and modification of SAP into targeted brain delivery vehicles. (A) SAP as a PPII helix. Hydrophilic residues (R) are shown in blue while hydrophobic residues (V and L) are shown in yellow. (B) Simplification of the defined faces presented by SAP demonstrating the defined structure of the PPII helix. (C) Mutation of similar (V and V) or alternative residues (V and L; as shown in figure) on the SAP scaffold enables RMT ligands to be appended on the same or opposite face of the PPII helix in a bivalent format.

Here we present the design, synthesis and validation of a diverse shuttle library to identify key physicochemical properties for transcytosis. We based our approach on two essential modular components to develop the chemical tool kit: (i) sweet-arrow-peptide (SAP), an isolated sequence derived from the N-terminal proline rich domain of γ-zein, and an innate CPP with a defined PPII secondary structure to act as a scaffold,^2^,^7^ and; (ii) a variety of validated RMT ligands to enable targeted delivery,^8^,^9^ Fig. 1 and Table 1.

**Table 1:** Sequences and target of peptide ligands used for delivery vehicle construction. Peptides were synthesised with either FAM conjugated to the N-terminus or azidolysine (K(N_3_)) for use as a control or for conjugation to SAP respectively

Peptide	Sequence	RMT target
SAP	VRLPPPVRLPPPVRLPPP	NA
TfRL1	THRPPMWSPVWP	TfR
TfRL2	HAIYPRH	TfR
APep2	TFFYGGSRGKRNNFKTEEY	LRP1

It was anticipated that uptake at the BBB could be probed through exploring ligand type (*i.e.* targeted receptor, affinity) and arrangement (valency, position) of the ligands fused to the scaffold, since previous studies have indicated that brain exposure is directly affected by these parameters.^3^,^5^,^6^,^10^,^11^ We provide compelling evidence that uptake and brain trafficking can be improved by combining AMT and RMT motifs on a single shuttle and that better understanding of receptor mediated trafficking within the brain endothelium is required at an individual and ligand-by-ligand basis, with ligand type, number and position effecting permeability in endothelial cells.

## Results and discussion

### Design and synthesis of brain delivery shuttles

SAP, the core of our delivery vehicles, is formed from a short repeating sequence of (VRLPPP)_3_ that is readily accessible in high yield through general automated solid-phase peptide synthesis (SPPS). It is noteworthy to mention that SAP retains a PPII conformation if 50% of the sequence remains as proline, forming a left-handed helix of 3.0 residues per turn, which affords three distinct ‘faces’ in aqueous solution, Fig. 1B.^7^,^12^,^13^ Consequently, spatial parameters of the BBB shuttle could be readily studied through simple mutation of valine, arginine, or leucine amino acids to orthogonal residues at specific sites. For this, we included lysine (K) or propargylglycine (X) on the PPII scaffold to conjugate relevant RMT motifs through amide bond forming and copper alkyne–azide chemistry (CuAAC) conjugations respectively. The full scaffold library we generated is shown in Table 2. Mutations to the SAP primary sequence follow standard convention and, when conjugated to the specified ligand, the attachment point on the scaffold is given in brackets and in order of conjugation. All peptide scaffolds were made with the chemically inert C-terminal amide in place of a carboxylic acid and FAM was conjugated to the N-terminus as cargo and to allow *in vitro* study.

**Table 2:** Peptide scaffolds synthesised and used for delivery vehicle construction. Peptides were synthesised with FAM conjugated to the N-terminus. Monovalent PPII(R8K) incorporated K(ivdde) for continuation of peptide synthesis while PPII(L3X;L15K) and PPII(L3X;V13K) included both K(ivdde) and K(Boc). Mutated residues in the sequence are shown in bold

Peptide	Sequence	Conjugation
PPII(R8X)	VRLPPPV**X**LPPPVRLPPP	CuAAC
PPII(R8K)	VRLPPPV**K**LPPPVRLPPP	Amide
PPII(L3X)	VR**X**PPPVRLPPPVRLPPP	CuAAC
PPII(L15X)	VRLPPPVRLPPPVR**X**PPP	CuACC
PPII(L3X;L15X)	VR**X**PPPVRLPPPVR**X**PPP	CuAAC
PPII(L3X;V13X)	VR**X**PPPVRLPPP**X**RLPPP	CuAAC
PPII(L3X;L15K)	VR**X**PPPVRLPPPVR**K**PPP	Amide, CuAAC
PPII(L3X;V13K)	VR**X**PPPVRLPPP**K**RLPPP	Amide, CuAAC

To enable targeted brain delivery, we chose to functionalise our scaffold with established ligands that engage the transferrin receptor (TfR) and low-density lipoprotein-receptor related protein (LRP1). The relative abundance of these receptors on brain endothelial, alongside their high transport capacity, made them ideal targets for RMT mediated delivery and both have been actively explored in research.^8^,^14^–^16^ We focused on three main peptide ligands for these receptors, as shown in Table 1. Both TfRL1 and TfRL2 have been shown to interact with TfR at alternative sites compared to transferrin (Tf), and have either nanomolar (TfRL1; 15.0 nM) or micromolar affinity (TfRL2; 440 μM) towards the receptor.^9^ Furthermore, branched BBB-shuttles incorporating dimers of TfRL1 have recently shown a non-linear increase in uptake within cellular models of the BBB.^11^ Alternatively, Angiopep2 (APep2) displays a high transcytosis potential mediated by LRP1.^8^ While less is known about trafficking of LRP1, APep2-conjugates have shown demonstrable success in clinical trials of neurological disease models.^8^ Peptide ligands were synthesised by general SPPS on rink-amide resin (see Table S1† for characterisation data and yields). It is important to note that the stability of the peptide vehicles was not considered in this proof-of-concept screen. Recent investigations with *retro-enantio* sequences of both TfRL1 and APep2 have shown that metabolic limitations can be overcome without the loss of function, offering a plausible solution to degradation.^14^,^17^ This is also apparent for the d-amino acid analog of SAP.^18^

### Chemoselective synthesis of brain delivery shuttles

**Scheme 1:**
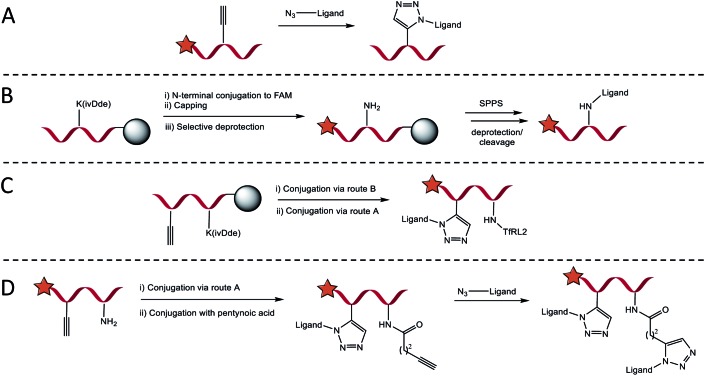
General approach for introducing RMT ligands on the SAP backbone. (A) Introduction of RMT ligands (up to two), with azidolysine incorporated as the final amino acid in their sequence, by CuAAC when proparglyglycine is included in the SAP sequence. (B) Route for introducing ligands by amide bond forming reaction with lysine. Following SPPS and N-terminal FAM conjugation, orthogonally protected lysine is selectively deprotected on resin to enable continuation of SPPS on the exposed ε-amino group. (C) Trifunctional vehicles incorporating TfRL2 on lysine can be formed by combining condensation and CuAAC routes. (D) Alternatively, trivalent vehicles can be formed in higher yield by performing CuAAC with an N-terminally capped ligand followed by conjugation of pentynoic acid on the ε-amino group of lysine. An alternative RMT ligand is then introduced by a secondary CuAAC reaction.

Four conjugation approaches were used to decorate the scaffold in various formats, Scheme 1A–D. Solution phase CuAAC chemistry with microwave heating was most effective for affording PPII-ligand conjugates in either monovalent or bivalent formats that included identical ligands (Scheme 1A). Alternatively, for the attachment of orthogonal ligands to the scaffold, TfRL2 was first incorporated *via* continuation of Fmoc/*t*Bu SPPS on the lysine side chain of PPII scaffolds, as generalised in Scheme 1B. Following FAM conjugation, unreactive N-terminal PPII was capped to ensure no unwanted reactions, before the lysine protecting group (ivDde) was selectively removed on resin. Resin was re-submitted to automated SPPS to afford the product following deprotection and cleavage. If propargylglycine (X) was also incorporated on the scaffold, bivalent conjugates with alternative ligands were afforded through subsequent reaction by CuAAC (Scheme 1C). While this approach afforded conjugates in high yield, it was somewhat limited, since conjugates of both TfRL1 and APep2 were unattainable through continuation of SPPS on resin. To overcome this limitation, CuAAC could be used to attach one ligand to the PPII scaffold, which was followed by subsequent coupling of small molecule pentynoic acid to lysine under amide bonding forming reactions. A secondary CuAAC reaction afforded bivalent scaffolds with alternative ligands in moderate yield (Scheme 1D). As demonstrated here and elsewhere, CuAAC mediated convergence provided rapid access to versatile bioconjugates that was reproducible.^19^ Detailed characterisation data and yields for all conjugates are reported in Table S2.†

### Secondary structure determination of brain shuttles

**Fig. 2:**
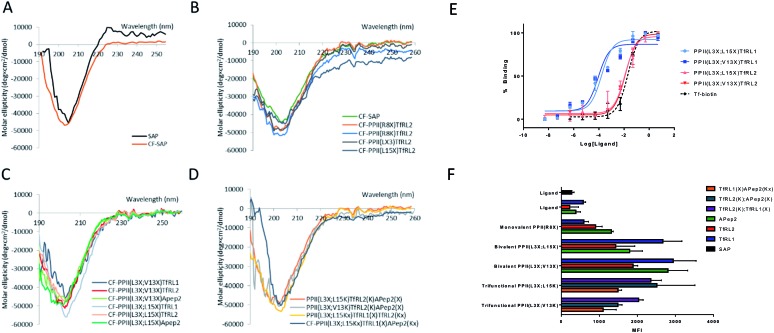
Properties and uptake capacities of modified SAP vehicle library. (A–D) CD spectra of a selection of FAM labelled delivery vehicles (50 μM) in phosphate buffer at pH 7. (A) Wild-type SAP (black) and N-terminally conjugated FAM SAP (CF-SAP; orange). (B) CF-SAP with monovalent vehicles, conjugated to TfRL2 in various positions on the SAP backbone or attached by different conjugation techniques. (C) Bivalent vehicles with ligands incorporated on the same or opposite face of SAP. (D) Trivalent vehicles, incorporating alternative AMT ligands on SAP backbone. (E) ELISA displays the apparent binding affinity (EC_50_) to TfR for Tf (black) or bivalent TfRL1 (blue) and TfRL2 (red) vehicles where ligands are either appended to the same (light) or opposite face (dark). (F) Uptake capacity of FAM labelled vehicles in bEnd.3 cells by flow cytometry. Extracellular fluorescence was quenched by addition of trypan blue. MFI = mean fluorescent intensity.

We determined structural integrity of the PPII helix by circular dichroism (CD) spectroscopy, where spectra of unmodified SAP and the N-terminal FITC-conjugated analogue were collected as controls, Fig. 2A. Importantly, the results suggest preservation of the secondary PPII structure following modification to the backbone since all peptides demonstrated strong absorption at 203 nm, Fig. 2B–D. Specifically, PPII helicity was insensitive to the conjugate linkage chemistry, ligand type and number of additions to the PPII scaffold.

### *In vitro* binding capacity of shuttles towards TfR

To evaluate functionality of the conjugated peptides, *in vitro* analyses were performed. TfR binding was assessed through an ELISA format, whereby FAM-conjugated shuttles were serially diluted and added to plates that were coated with TfR protein. FAM-labelled vehicles were detected by an anti-FAM HRP-conjugated antibody. As predicted, TfRL1-vehicles contained a higher affinity towards the TfR than the equivalent vehicle incorporating TfRL2 (PPII(L3X;L15X)TfRL1: 0.26 ± 0.17 nM; PPII(L3X;V13X)TfRL1: 0.31 ± 0.26; *cf.* PPII(L3X;L15X)TfRL2: 11.8 ± 1.11 nM; PPII(L3X;V13X)TfRL2: 12.7 ± 1.37), Fig. 2E.^9^ Notably, TfRL2 bivalent shuttles contained a similar affinity towards TfR compared to Tf-biotin when assayed in a similar format (EC_50_: 20.9 ± 2.1 nM). It is assumed that an avidity effect is demonstrated by the bivalent vehicles since monovalent TfR-targeting shuttles contained too low-affinity towards TfR to be detected. Further, positioning of the ligand did not affect TfR binding affinity since scaffolds that incorporated ligands on the same face afforded affinity values similar to those with ligands fused on different sides.

### Evaluation of the BBB-shuttle properties of the conjugates in brain endothelial cells

Having demonstrated preservation of receptor binding, we next used bEnd.3 cells to screen the uptake capacity of the shuttle library by flow cytometry. We determined peptide uptake and internalisation in a confluent monolayer of bEnd.3 cells which is an immortalised mouse brain endothelial cell line that has similar characteristics to the BBB. TfR is an established receptor for clathrin dependent uptake of Tf, favoured in an iron bound form when at physiological pH (*i.e.* holo-Tf).^20^ Flow cytometry (FC) and microscopy data obtained with human derived AlexaFluor 647-conjugated Tf (Tf-A647) confirmed functional, membrane resident TfR (see Fig. S1 and S2†).

### Evaluation of the internalisation capacity in brain endothelial cells

For library screening, cells were exposed to equal concentrations (500 nM) of FAM labelled molecules in media for 3 h to allow binding, internalisation and sorting, and external FAM fluorescence was quenched by addition of trypan blue.^21^ Low temperature was shown to inhibit cellular uptake of the vehicles (Fig. S3†), demonstrating an energy-driven internalisation process. Furthermore, using DAPI as a viability indicator, no cytotoxicity of the vehicles was demonstrated during these experiments and over a concentration range of up to 100 μM, where viability remained over 98% (Fig. S4†).

It can be seen from Fig. 2F that, in general, brain shuttles contained heightened, non-additive, uptake compared to their unconjugated counterparts, implying a synergistic effect for uptake. TfRL1 demonstrated the highest capacity for endocytosis when incubated alone, however appreciable difference was shown for the monovalent vehicle in this format (TfRL1 *cf.* PPII(R8X)TfR1 *P* = 0.798; TfRL2 *cf.* PPII(R8X)TfR2 *P* = 0.018; APep2 *cf.* PPII(R8X)APep2 *P* = 0.002). Notably, when arranged in a bivalent format, TfRL1 modified vehicles showed a dramatic increase in cellular internalisation that exceeded additive contributions. Interestingly, endocytosis of TfR-targeting bivalent vehicles was not significantly influenced by its positional arrangement on the scaffold (PPII(L3X;L15X)TfRL1 *cf.* PPII(L3X;V13X)TfRL1 *P* = 0.408; PPII(L3X;L15X)TfRL2 *cf.* PPII(L3X;V13X)TfRL2 *P* = 0.050), compared to those targeting LRP-2 which favoured ligands attached to opposite faces of the scaffold (PPII(L3X;L15X)APep2 *cf.* PPII(L3X;V13X)APep2 *P* = 0.0059). This phenomenon could be attributed to increased steric strain of the larger APep2 peptide when incorporated on the same face, or possibly linked to the ligands ability to influence receptor clustering on the extracellular membrane. Again, higher uptake was demonstrated for scaffolds that combined alternative transcytosis ligands on the same molecule compared to individual ligands. However, uptake was not improved compared to bivalent scaffolds containing identical ligands.

### Intracellular location of BBB shuttles

**Fig. 3:**
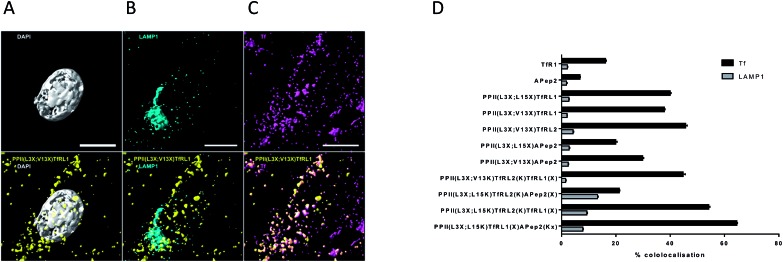
Cellular location of FAM labelled PPII vehicles and RMT ligands in bEnd.3 cells. (A–C) Show 3D segmentation of fluorescent signal for bivalent vehicle PPII(L3X;V13X)TfRL1 (yellow, all images), showing low association with (A) the nucleus (grey), (B) LAMP1 (cyan), but high association with (C) Tf-A647 (magenta). Cultures were fixed in 4% PFA, permeabilised and immunostained with an antibody for the late endosome/lysosome marker LAMP1 as described in the ESI.† Scale bar represents 10 μM. (D) Colocalisation of vehicles with Tf-A647 (early and recycling endosomes; black) or LAMP1 (lysosomes; grey). Data shown is mean ± SEM of the percentage of 3D spatial overlap between individual vehicle objects and Tf or LAMP1 objects.

To obtain information on the intracellular fate of the shuttles, bEnd.3 cells were pulsed with bivalent vehicles, Tf-A647 and unconjugated TfRL1 and APep2 ligands for 3 h, unbound compounds were removed and cells fixed and stained for immunofluorescence microscopy. Images were processed as described in Fig. S5.† Fig. 3A–C shows colocalisation of PPII(L3X;V13X)TfRL1 with DAPI, Tf-A647 or LAMP1 respectively and represents a typical image generated from the screen following deconvolution and segmentation. It can be seen from Fig. 3D that all vehicles gave highest colocalisation with Tf-A647 as opposed to LAMP1, indicating uptake into endosomal vesicles with minimal degradation through lysosomal sorting. In addition, Tf-A647 colocalisation values were higher for vehicles compared to the individual RMT ligand components. Notably, the degree of Tf-A647 and LAMP1 colocalisation was comparable for TfRL1 vehicles incorporating this ligand on the same or opposite face. In addition, Tf-A647 colocalisation for bivalent TfRL1 vehicles and PPII(L3X;L15X)TfRL2 was also comparable. However, increased levels of LAMP1 colocalisation was observed for TfRL2 bivalent vehicle, indicating that the higher affinity TfR ligand is more efficient for non-degradative cellular uptake in this system. Colocalisation of APep2 bivalent vehicles with Tf-A647 were affected by the position of the ligand, demonstrating a similar trend to that shown with endocytotic capacity, Fig. 2F. Trivalent vehicles decorated with TfR ligands demonstrated similar colocalisation with Tf-A647 to those in a bivalent format. Whilst vehicle PPII(L3X;L15K)TfRL2(K)TfRL1(X) demonstrated highest Tf-A647 colocalisation, it also had heightened association in the lysosome. Notably, PPII(L3X;L15K)TfRL2(K)APep2(X) had low Tf-A647 colocalisation and the highest LAMP1 association out of the vehicles screened. This could imply that the ligands are incompatible when combined on the same vehicle, or that endocytosis is supported through a different mechanism of uptake. Alternatively, trivalent vehicle PPII(L3X;L15K)TfRL1(X)APep2(Kx) gave high Tf-A647 colocalisation and moderate LAMP1 association.

### Permeability experiments

To study transcytosis potential of the vehicles *in vitro* we optimised a BBB model using a 3D-transwell format. We found that a bEnd.3/mesenchymal stem cell (MSC) co-culture reproducibly afforded the highest resistance to paracellular diffusion through assessment with transepithelial electrical resistance measurements (TEER) and small molecule permeability studies (Fig. S6†). This is similar to results shown by others.^16^ Immunostaining for TJ protein ZO-1 in the bEnd.3 cell line confirmed its presence and trafficking towards the cell junctions (Fig. S7†). While this indicates that an adequate and reproducible barrier was formed from the bEnd.3/MSC co-culture model, we found that it was of paramount importance to distinguish transcytosis from the expected background of paracellular flow since others have reported issues distinguishing these values.^22^

### Optimisation and adaptation of BBB *in vitro* model

Often *in vitro* assays of the BBB overlook contributions of paracellular flux that can lead to overestimations of brain exposure, and offer no valid comparison between the individual molecules being screened. We believed that without a quantifiable probe for paracellular diffusion, it would be difficult to accurately screen the transcytosis capacity of our vehicles.

**Fig. 4:**
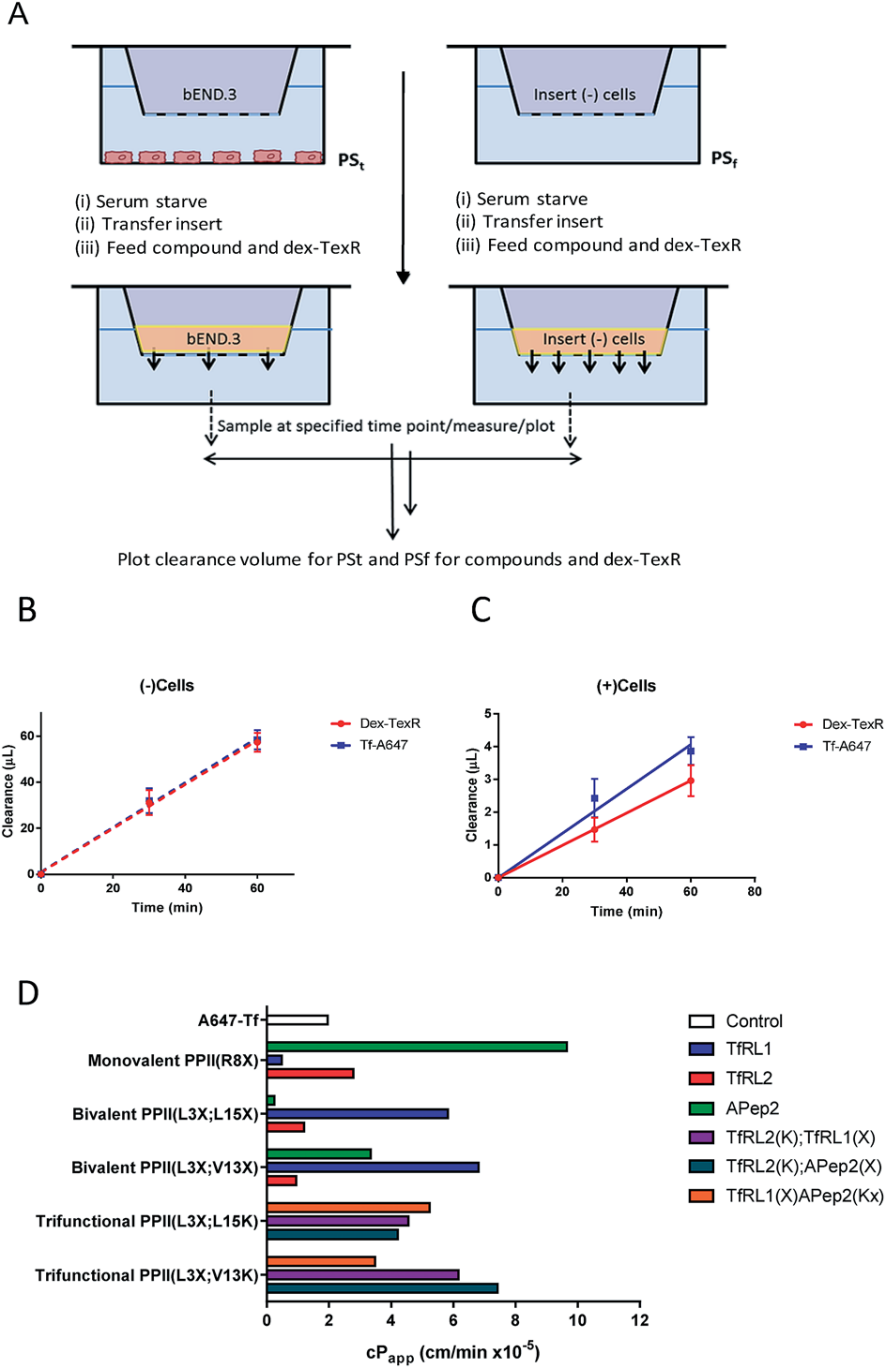
Modified workflow and results for determining the _c_*P*_app_ of a macromolecule, accounting for contributions made by paracellular diffusion across an *in vitro* barrier. (A) Experimental workflow demonstrating that compounds are co-incubated with dex-TexR either in the presence of cell monolayer (PS_t_; (+)cells) or with filters alone (PS_f_; (–)cells) to obtain *P*_app_ values through generation of clearance plots. (B–C) Clearance plots obtained by incubation of Tf-A647 or dex-TexR with filters alone (B; PS_f_) or with cells (C; PS_t_). Clearance is significantly lowered in the presence of cells. Division of PS_t_/PS_f_ for dextran generates the transport ratio (TR). Expected paracellular contribution of the vehicle is obtained by multiplying TR by the *P*_app_ of the compound of interest without cells. _c_*P*_app_ values are obtained by subtracting this value from *P*_app_ of compound with cells. Key: red = dex-TexR; blue = Tf-A647; dashed lines = filter insert only; solid lines = transwell culture (D) _c_*P*_app_ values for delivery vehicles screened in the transcytosis assay.

It seemed plausible that a similar sized marker (such as TexasRed labelled dextran (dex-TexR; 3000 g mol^–1^)) that is incapable of AMT or RMT uptake could serve as an internal standard to quantify paracellular contribution of the vehicles. As exemplified in Fig. 4A, experiments are performed in parallel with probes (both dex-TexR and FAM-PPII vehicle or Tf-A647) fed to insets containing either the cell monolayer (PS_t_) or filter alone (PS_f_). The corresponding *P*_app_ for dex-TexR in both scenarios is first calculated and the extent in which movement is reduced by presence of the monolayer (defined here as the transport ratio (TR)) is used to determine the expected paracellular diffusion rate of the individual vehicles. We employed Tf-A647 and a similarly sized TexasRed labelled dextran (dex-TexR; 70 000 g mol^–1^) as a positive control for transcytosis. Typical clearance profiles when compounds are incubated with and without cells, are shown in Fig. 4B–C. As expected, due to their similar molecular weight, both dex-TexR and Tf-A647 afforded comparable clearance when incubated without cells (PS_f_). When incubated in the presence of cells, dex-TexR was detected in the basolateral compartment indicating background paracellular flux. However, Tf-A647 was cleared at a faster rate in comparison, confirming transcytosis of the molecule.

Corrected values were subsequently calculated to internally rank the transcytosis capacity of BBB-shuttles. In addition, to verify validity of the model, a selection of high permeability vehicles were selected for further studies at low temperature, where uptake and transcytosis should be inhibited. In agreement, shuttles showed permeability similar to dex-TexR (3000 g mol^–1^), demonstrating negligible true transcytosis in this condition (Fig. S8†).

### Permeability screen of BBB shuttles

Our screen shows that the majority of functionalised vehicles had higher rates of permeability than Tf-A647 with PPII(R8X)TfRL1, PPII(L3X;L15X)APep2 and both bivalent TfRL2 vehicles being the exception, Fig. 4D. TfRL1 modified vehicles preferred a bivalent format (PPII(L3X;V13X)TfRL1: 3.5-fold increase relative to Tf-A647; PPII(L3X;L15X)TfRL1: 3.0-fold), whereas TfRL2 and APep2 monovalent vehicles experienced higher levels of basal transport (PPII(R8X)TfRL2: 1.4-fold; PPII(R8X)APep2: 4.9-fold), with the latter vehicle showcasing the best permeability of those screened. In agreement with the endocytosis and microscopy studies, TfR targeting bivalent vehicles showed a moderate or no clear positional preference for ligands attached to alternative faces, whereas Apep2 highly favoured ligands attached to alternative faces of the scaffold with PPII(L3X;L15X)Apep2 showing limited transcytosis. In agreement with the microscopy data, trivalent vehicles where both ligands are directed towards the TfR demonstrated improved transcytosis when ligands were incorporated on opposite sides of the scaffold. In addition, the data implies that affinity towards the TfR does not govern transport ability for our peptidic BBB shuttles since trivalent vehicles combining high and low affinity ligands showed lower permeability than the corresponding bivalent high affinity TfRL1 vehicles.

Whilst vehicle PPII(L3X;V13K)TfRL2(K);APep2(X) gave the highest level of transcytosis for a highly functionalised shuttle (3.8-fold increase relative to Tf-A647), the increased permeability may be attributed to the ligands interacting independently with their receptors, since both ligands enjoyed higher transport in a monovalent form. In line with this hypothesis, transcytosis for PPII(L3X;L15K)TfRL2(K);APep2(X) was lowest for trifunctional vehicles with ligands merged to the same face (1.8-fold), implying that the ligands are not working in synergy. This result is in contrast to when ligand TfRL1 and APep2 are combined, which showed improved permeability when ligands are arranged on the same face.

## Conclusions

Here we report the first use of CPP SAP as a scaffold for developing targeted BBB penetrable shuttles, which constitutes one of the most restrictive barriers in the body. In this regard, we designed a versatile vehicle library by a convergent approach, strategically introducing mutations within the sequence to graft RMT ligands in a selective manor using either amide bond forming or CuAAC mediated reactions. As noted elsewhere, CuAAC provided a superior and flexible reaction for these modifications. Notably, SAP retained a helical PPII structure after modification, and vehicles screened in biologically relevant assays demonstrated uptake and trafficking of cargo at the BBB. It was shown that AMT and RMT motifs worked in synergy to encourage cellular uptake, with certain molecular characteristics such as affinity, position and valency influencing both uptake and transcytosis for individual ligands. Notably, the majority of vehicles screened demonstrated heightened transcytosis rates compared to Tf in a BBB model. Here we believe the small size of the peptide conjugates, compared to Tf and other macromolecule shuttles, afford them an advantage for targeted transcytosis due to higher diffusion rates. Within our permeability screening campaign, dex-TexR was successfully included as an internal standard for quantifying paracellular and non-specific movement. Consequently, the results presented demonstrate that PPII derived shuttles represent novel, exciting and promising classes of bioconjugates for enhancing uptake at the BBB. The flexibility of the screening approach could be readily adopted to investigate other ligands for AMT and RMT uptake at the BBB to validate and identify optimal ligands and shuttles for delivery.

## Conflicts of interest

There are no conflicts of interest to declare.

## Supplementary Material

Supplementary information
