# Supplementary material for: Chemical biology tools for probing transcytosis at the blood–brain barrier
Source: Chem Sci. 2019 Oct 8;10(46):10772–8. doi: 10.1039/c9sc04024b (PMC7006505; doi:10.1039/c9sc04024b)
Supplement: Supplementary file 1 [file SC-010-C9SC04024B-s001.pdf]

Electronic Supplementary Material (ESI) for Chemical Science.
This journal is © The Royal Society of Chemistry 2019

# Precise biochemical tools for probing transcytosis at the Blood-Brain Barrier

Rhiannon Beard[1]; David C. A. Gaboriau[2]; Antony Gee[3]; Edward W. Tate[1,4]

*[1]Department of Chemistry, Imperial College London, Exhibition Road, London; [2]Facility for Imaging by Light Microscopy, Sir Alexander Fleming Building, Imperial College London, Exhibition Road, London; [3]Division of Imaging Sciences, The Rayne Institute, 4th Floor, Lambeth Wing, St Thomas' Hospital, London; [4]Francis Crick Institute, 1 Midland Road, London*

# SUPPORTING INFORMATION

Table of contents

**Part 1. Materials and Methods**

**Part 2. Supporting Figures**

Figure S1. FC data obtained with Tf-A647 in bEND.3 cells

Figure S2. Wild-field (WF) images of Tf-A647 (25 µg/ml) endocytosis following 3 h incubation at 37 °C in bEND.3 cells

Figure S3. Temperature dependence for uptake of bivalent scaffolds (0.5 µM) in media by FC

Figure S4. Cytotoxicity of selection of vehicles over a concentration range by FC

Figure S5. Experimental TEER and $P_{app}$ values for paracellular diffusion marker LY in various *in vitro* blood-brain barrier models

Figure S6. Imaging of BBB vehicles in bEND.3 cells with Tf-A647, LAMP1 and DAPI for quantification of colocalisation.

Figure S7. Immunofluorescent staining of confluent bEND.3 cells for TJ protein ZO-1 (red) and nucleus (blue) by confocal microscopy

Figure S8. Clearance plots for a selection of FAM-labelled vehicles at 4 °C

**Part 3. UV and Mass Spectrometry characterisation data for BBB shuttles**

## Part 1. Materials and Methods

### General Considerations

General laboratory chemicals, solvents and biological components for cell culture were obtained from Sigma-Aldrich Chemical Co., Novabiochem UK, Merck, VWR or Invitrogen and used without further purification. Gases were from BOC. Solutions and buffers were prepared with Ultra-pure water obtained from a Millipore Elix Q-guard purification system.

Microwave-assisted reactions were conducted using an Initiator Classic microwave (Biotage). UV-vis analysis (for measurement of the A280 or A490 of a peptide solution containing tyrosine, tryptophan and phenylalanine amino acids residues or FAM respectively) was carried out on a NanoDrop apparatus (Labtech). FC experiments were performed using a Fortessa flow cytometer (BD bioscience), running FlowJo software (version 10). Fluorescence measurements, for $P_{app}$ determination, were performed in black 96-well plates (Greiner bio-one; #655076), and measured with a SpectraMax M2E Microplate reader (Molecular Devices). Images for colocalisation analysis were acquired with a Zeiss Axio Observer microscope with a 63x/1.40 Plan Apochromat objective, ZEN Blue Pro software (Zeiss), a Hamamatsu Flash 4.0 camera and a Lumencor Spectra X LED light source.

### Analytical and purification methods

Purification of synthesised crude peptides and analysis was performed on a Waters LC-MS system (Waters 2767 autosampler for samples injection and collection; Waters 515 HPLC pump to deliver the mobile phase to the source; Waters 3100 mass spectrometer with ESI; and, Waters 2998 Photodiode Array (detection at 200–600 nm)), equipped with XBridge $C_{18}$ reverse-phase columns with dimensions 4.6 mm × 100 mm for analytical and 19 mm × 100 mm for preparative runs. Solvents were degassed with helium and supplemented with 0.1 % formic acid prior to use. General methods are given below. Flow rates were either 1.2 mL/min or

20 mL/min for analytical or semi-preparative runs respectively, using water supplemented with 0.1% formic acid and MeOH as solvents.

*LC-MS Analytical gradient:* 5% to 98% MeOH in $H_2O$ over 10 min, 98% MeOH was held for 2 min, MeOH was reduced from 98% to 5% over 1 min, and held at 5% until 18 min. *LC-MS semi-preparative gradient:* 5% to 25% MeOH in $H_2O$ over 1 min, then increased to 75% MeOH over 10 min. MeOH was further increased to 98% over 1 min, held at 98% MeOH for 1 min, then reduced to 5% MeOH over 1 min where it was held for 4 min (18 min total).

**Solid phase peptide synthesis**

Peptides were synthesised using automated solid-phase peptide synthesis on a ResPep SL apparatus (Intavis) using the supplied Multipep software. Following resin cleavage/deprotection and precipitation, peptides were pelleted at 4 °C using a Jouan BR4 centrifuge. Following purification by LC-MS, solvent was removed with a centrifugal evaporator (Genevac), and lyophilisation of peptides were done with an Alpha 1-4 freeze dryer (Christ, Osterode am Harz Germany). All peptides were made with Rink amide Tentagel resin (Rapp Polymere; 0.71 mmol/g) to afford a *C*-terminal amide group.

**General methods for solid phase peptide synthesis**

Protocols for reaction or manipulation of resin-bound peptide in this section correspond to a 20 µmol scale, (i.e. one column in the automated synthesiser), but were adapted for scale up when necessary.

*Solid Phase Peptide Synthesis*

Synthesis was carried out in peptide synthesis grade DMF. Resin was swelled in DMF for 30 min before synthesis. Subsequent steps were conducted automatically. N-α-amino Fmoc groups were deprotected using 20 % (v/v) piperidine in DMF (400 µL, 2 × 5 min). The Fmoc-

protected amino acid (100 μmol, 5.0 eq.; 200 μL of 0.5 M stock solution in NMP) for coupling was preactivated with HBTU (95 μmol, 4.75 eq.; 190 μL of 0.5 M stock solution in NMP) and NMM (200 μmol, 10 eq.; 50 μL of 4 M stock solution in NMP). Coupling was allowed to take place over 30-55 min and amino acids were 'double-coupled' (i.e. the coupling step was repeated). To prevent deletion sequences, any unreacted N-terminal amines were acetylated with 'capping mixture' (400 μL of 5 % (v/v) $Ac_2O$ in DMF) for 10 min. The resin was washed with DMF (3 × 1 mL) between the deprotection, coupling and acylation steps. The typical cycle was repeated until the final (*N*-terminal) amino acid coupling, when *N*-α-Fmoc deprotection at the final residue undertaken under usual conditions.

For any modifications to the resin bound crude peptides after synthesis (as listed below), resin was transferred to a fritted 5 mL syringe, washed (DMF 3 × 1 mL, DCM 3 × 1 mL, DMF 3 × 1 mL) and swelled in DMF, for a minimum of 1 h with shaking, before subjection to the required conditions.

*N-terminal acetylation*

1 mL of solution of 'capping mixture' supplemented with DIPEA (5 % (v/v)) was added to the crude peptide, and the resulting slurry was agitated for 30 min. Solution was removed by suction, washed (DMF 3 × 1 mL, DCM 3 × 1 mL) and the process repeated.

*N-terminal coupling to FAM*

FAM (100 μmol, 5 eq.) was dissolved in 0.5 mL DMF and DIPEA (100 μmol, 5 eq.). HATU (98 μmol, 4.9 eq.) was added, and the mixture agitated for 5 min. After this time the preactivated FAM mixture was added to a slurry of resin bound peptide in 0.5 mL DMF and DIPEA (20 μmol, 1 eq.) and shaken vigorously for 1.5 h. The solution was removed by suction, the resin washed and the process was repeated.

*Removal of ivdde orthogonal protecting group for lysine*

Where applicable, ivdde protected lysine was selectively removed by addition of hydrazine monohydrate (4 % in DMF; 3 × resin volume) for 1 h with shaking. The solution was removed by suction, resin washed, and the SPPS process repeated.

*Deprotection, cleavage and purification of peptides*

Following synthesis or modification, peptides were washed several times (3 × 1 mL DMF, 3 × 1 mL DCM, 3 × 1 mL MeOH, 3 × 1 mL $Et_2O$) and dried overnight in a desiccator. For the deprotection and cleavage of peptides containing cysteine and methionine, a mixture of 1.5 mL TFA:$H_2O$:DTT:TIS (94:2.5:2.5:1) was added to resin bound peptide, unless otherwise stated. For the cleavage and deprotection of all other peptides, a mixture of TFA:$H_2O$:TIS (95:2.5:2.5) was used. Crude peptides were precipitated using ice cold TBME, centrifuged at 4000 rpm for 15 min at 4 $^{\circ}$C, and the supernatant discarded. The remaining peptides were washed with a fresh aliquot of TBME and the process repeated. Precipitate was dried in a desiccator over silica gel to yield off white solids which were dissolved in a $H_2O$:MeOH mixture for purification by RP LC-MS. Following purification, fractions containing pure peptide were combined and concentrated in the Genevac. Subsequently, pure peptides were re-dissolved in water and freeze-dried overnight.

**Chemical conjugation protocols for vehicle library synthesis**

Conjugations were performed between two or three components, namely (i) RMT ligands with the complementary modification towards the *N*-terminus, and; (ii) PPII helix scaffolds.

*Amide bond conjugation of RMT ligands using orthogonally protected lysine*

Scaffold primary sequences, incorporating K(ivdde) in the relevant positions, were synthesised and resins retrieved. FAM was “doubly coupled” on the *N*-terminal, after which any unreacted

amine was acetylated. The ivdde group was removed and resin bound peptide (10 µmol; i.e. half of the original resin) was placed back on the synthesiser for introduction of the required peptide ligand on the ε-amino group of lysine by general automated Fmoc SPPS.

*Amide bond conjugation of pentynoic acid to lysine*

The procedure reported below is for single addition to a lysine containing PPII scaffold, conjugated to FAM on the N-terminal. When necessary, the procedure was scaled up accordingly. Pentynoic acid (1.5 eq.) was preactivated by dropwise addition of a solution of HATU (1.4 eq.) and DIPEA (3 eq.) in 0.5 mL DMF. The mixture was agitated for 5 min then added to a solution of PPII scaffold to afford a peptide concentration of 1 mg/mL in DMF. The mixture was agitated for 1.5 h, after which time the solution was diluted 5-fold with water and purified by LCMS. The conjugate was then subjected to CuAAC as outlined below.

*Conjugations via CuAAC*

All conjugations of RMT ligands, modified by addition of azido lysine, were performed in solution phase. Reactions were performed on a 1 mg scale of PPII scaffold and scaled up accordingly.

For single conjugations, $CuSO_4$ (10 eq.), TCEP (10 eq.), TBTA (0.1 eq.) and DIPEA (2 eq.) was added sequentially to a solution of RMT ligand azide (1.5 eq.) in water. A solution of PPII scaffold (100 µL of 10 mg/ml solution in water) was added and the resulting mixture made to a peptide concentration of 0.5 mg/mL. The solution was heated with stirring for 3 h at 45 °C in the microwave, after which time the solution was concentrated in the genevac. The precipitate was re-suspended in a mixture of water and methanol, desalted on a Sep-Pak® Plus $tC_{18}$ column (Waters, WAT036800), and purified by LC-MS. Double conjugations followed a similar procedure but required an increased amount of RMT ligand (3.0 eq.), as well as double the amount of reagents ($CuSO_4$, TCEP, TBTA and DIPEA).

## Characterisation of peptides

*Circular Dichroism*

CD spectra were recorded using a Chirascan spectropolarimeter (Applied Photophysics). 200 µL of peptide solution (50 µM) in phosphate buffer (0.01 M, pH 7.4) were placed in a quartz cuvette (1 mm path length), and the CD spectrum recorded between 190–260 nm at a spectral bandwidth of 1 nm with step resolution of 1 nm and 1 s per step at 25 °C. Each spectrum represents an average of three scans.

*Mass spectrometry characterisation and yields for peptides*

Analysis of all peptides shown below in Tables S1 and S2 were performed with a Waters LC-MS system using the analytical method previously reported.

| Peptide | MW (Da) | $R_t$ (min) | ES peaks (m/z) | Yield (%) |
|---|---|---|---|---|
| FAM-TfRL1 | 1848 | 12.3 | 1849.3 (m+1), 925 (m/2+1) | 12 |
| FAM-TfRL2 | 1250 | 8.7 | 1251.5 (m+1), 626.0 (m/2+1) | 19 |
| FAM-APep2 | 2660 | 10.2 | 1330.6 (m/2+1), 887.4 (m/3+1) | 15 |
| K($N_3$)TfR1 | 1645 | 10.9 | 1644.9 (m+1), 823.1 (m/2+1) | 20 |
| K($N_3$)TfR2 | 1046 | 7.8 | 1047.7 (m+1), 524.5 (m/2+1) | 27 |
| K($N_3$)APep2 | 2455 | 9.5 | 1128.3 (m/2+1), 819.1 (m/3+1) | 9 |

**Table S1 Characterisation data for RMT ligands used for development of the delivery vehicle library.**

| Vehicle | MW (Da) | $R_t$ (min) | ES peaks (m/z) | Yield (%) |
|---|---|---|---|---|
| (R8X)TfRL1 | 3939 | 11.4 | 985.8 (m/4+1) , 788.8 (m/5+1), 660.1 (m/6+1) | 60 |
| (R8X)TfRL2 | 3340 | 10.4 | 1114.7 (m/3+1), 836.1 (m/4+1), 669.3 (m/5+1) | 64 |
| (R8X)APep2 | 4749 | 10.6 | 1188.1 (m/4+1), 951.3 (m/5+1), 792.6 (m/6+1) | 45 |
| (R8K)TfRL2 | 3200 | 10.4 | 1068.3 (m/3+1), 801.6 (m/4+1), 641.6 (m/5+1) | 12 |
| (L3X)TfRL2 | 3382 | 9.7 | 847.1 (m/3+1), 677.8 (m/4+1), 565.0 (m/5+1) | 55 |
| (L15X)TfRL2 | 3382 | 9.7 | 847.1 (m/3+1), 677.9 (m/4+1), 565.0 (m/5+1) | 58 |
| (L3X;L15X)TfRL1 | 5607 | 11.8 | 1122.7 (m/5+1) 935.6 (m/6+1), 802.3 (m/7+1) | 38 |
| (L3X;L15X)TfRL2 | 4408 | 10.2 | 883.4 (m/5+1), 736.5 (m/6+1), 631.4 (m/7+1), 552.6 (m/8+1) | 45 |
| (L3X;L15X)APep2 | 7228 | 10.9 | 1205.9 (m/6+1), 1033.8 (m/7+1), 904.7 (m/8+1), 804.2 (m/9+1) | 29 |
| (L3X; V13X)TfRL1 | 5619 | 12.1 | 1125.2 (m/5+1), 937.9 (m/6+1), 804.2 (m/7+1), 703.7 (m/8+1) | 41 |
| (L3X; V13X)TfRL2 | 4421 | 8.8 | 886.0 (m/5+1), 738.7 (m/6+1), 633.3 (m/7+1), 554.2 (m/8+1) | 50 |
| (L3X; V13X)APep2 | 7239 | 11.1 | 1208.2 (m/6+1), 1035.8 (m/7+1), 906.3 (m/8+1), 805.9 (m/9+1) | 39 |
| (L15K;L3X)TfRL2(K)TfRL1(X) | 4869 | 9.9 | 975.3 (m/5+1), 813.0 (m/6+1), 697.0 (m/7+1), 609.9 (m/8+1) | 31 |
| (L15K;L3X)TfRL2(K)APep2(X) | 5679 | 10.6 | 948.0 (m/6+1), 812.6 (m/7+1) | 29 |
| (L3X;V13K)TfRL2(K)TfRL1(X) | 4882 | 9.8 | 978 (m/5+1), 816 (m/6+1), 699 (m/7+1). 612 (m/8+1) | 45 |
| (L3X;V13K)TfRL2(K)APep2(X) | 5693 | 11.2 | 950.0 (m/6+1), 814.9 (m/7+1) | 34 |
| (L3X;V13K)TfRL1(X)APep2(Kx) | 6585 | 7.8 | 732.9 (m/9+1) | 21 |
| (L3K;L15X)TfRL1(X)APep2(Kx) | 6571 | 7.8 | 824.4 (m/8+1), 733.0 (m/9+1) | 39 |

**Table S2 Characterisation data for delivery vehicle conjugates.**

## Cell culture

All cells were maintained in an atmosphere of 5 % $CO_2$ and 95 % air using an New Brunswick Galaxy 1580R (Eppendorf). bEND.3 cells were routinely maintained in 10 $cm^3$ flasks, while the MSC cell line was cultured in 10 $cm^3$ flasks pre-coated with 2 µg/mL rat tail collagen type I (Sigma Aldrich). Wherever stated, cell washing relates to the addition and aspiration of PBS

(3 × 1 mL), and trypsinisation was conducted using a solution of 0.25 % (w/v) trypsin-EDTA solution (0.5 mL, 3 min, 37 °C, 5 % $CO_2$ incubation), unless otherwise specified. When required, a Scan Speed 1580R centrifuge (Labmode) was used to pellet cell suspensions. All tissue culture work was performed in sterilised Corning well plates, and cell based assays used DMEM media (ThermoFisher, #31053028) without FCS and phenol red, unless otherwise specified. bEND.3 cells were a gift from Rachael Brown (Kings College London), while MSC cells were a gift from the Battaglia group (University College London).

*bEND.3*

Cells were maintained in high glucose Dulbecco's Modified Eagle's Medium (DMEM) (ATCC #30-2002) supplemented with 10 % FCS. Cells were sub-cultured routinely using trypsin solution once 100 % confluence was achieved. For uptake and transcytosis studies, cells under passage number 30 were used. After this time, these cells may begin to lose their barrier functionality, as identified from an extensive study performed by Brown *et al.*[1]

*Mesenchymal stem cells (MSC)*

MSC, as surrogates for pericytes, were maintained in DMEM/F12 medium containing GlutaMAX and L-glutamine (2 mM) (ThermoFisher, #10565-018), supplemented with 10 % FCS and 5 µg/mL Gentamicin (SIGMA, G1397). Cultures were sub-cultured routinely using trypsin solution once 70-80 % confluence was achieved.

*Co-culture*

For all transcytosis assays, polyester filter inserts (Corning, #3460; 0.4 µm pore, 12 mm diameter, 10 µm thick) were used in 12-well cell culture plates. Optimum media volumes were calculated to be 950 µL and 475 µL for apical and basolateral chambers respectively. Both chambers were coated with rat tail collagen (5 $\mu g/cm^2$) for 2 h at room temperature prior to use. bEND.3 cells were seeded at a density of $2 \times 10^4$ cells/well onto insets, and grown to

confluent monolayers for 5 days. After this time, MSC cells were seeded at a density of 1.25 × $10^4$ cells/well on the basolateral chamber to generate endothelial-MSC co-cultures. Experiments with the co-culture model were performed 7 days after the bEND.3 cells were seeded. Empty filters, coated with collagen, were incubated with media containing 10 % FCS overnight before the assay was performed.

**In vitro Assays**

*Flow cytometry (FC) endocytosis assays*

bEND.3 cells were seeded at a density of 5 × $10^4$ cells/well in 12-well plates. Once cells were 100 % confluent, they were left for a further 3-5 days. Cells were washed and incubated with BBB vehicles (0.5 µM; total volume 1 mL) in DMEM, at either 37 °C or 4 °C for the specified time. After treatment, cells were washed, trypsinised (150 µL/well, 10 min), and re-suspended with 1 mL DMEM (containing 10 % FCS). Cell suspensions were transferred into falcon tubes and centrifuged (750 *g,* 5 min, 4 °C) to obtain a pellet. The supernatant was discarded, the pellet re-suspended with 0.5 mL media, centrifuged as before, and the supernatant removed. The cell pellet was re-suspended in DMEM containing 10 % FCS (450 µL/well) and transferred to FACS tubes, which were stored on ice. DAPI (Biolegend, 422801) (1 µg/ml) was added to differentiate the live/dead population, and fluorescence was measured immediately by FC. External fluorescence signal was then quenched by addition of TB (SIGMA, T6146) (10 µM) to the cell suspensions, which were vortexed (10 sec) and left to stand at room temperature for at least 3 min before the samples were re-measured for fluorescent levels by FC. When relevant, percentage quenching was calculated by the following equation: $((FL_{without} – FL_{with})/FL_{without}) \times 100$. Where: $FL_{without}$ is the mean fluorescence of the control without quenching solution and $FL_{with}$ is the mean fluorescence of sample following addition of the quenching solution.

*Statistical analysis*

Statistical analysis was carried out using the unpaired Student's t test algorithm on GraphPad Prism software. Findings were deemed significant if p values were less than 0.05.

*TEER measurements*

Cells were left to attach and media was changed 24 h before reading. Resistance measurements were taken with an EVOM voltohmmeter (World Precision Instruments). Electrodes were sterilised with EtOH:$H_2O$ (70:30) for 1.5 h prior to the measurements. Unit resistance was calculated by multiplying the area of the filter membrane (1.12 $cm^2$) by the resistance (Ω), and averaged for each sample (n=5). Background resistance corresponding to the uncoated insert was subtracted from each measurement.

*Transcytosis assays*

Inserts with and without cells were treated identically during the course of the experiment. On the day of the assay cells were serum starved for 1 h to deplete Tf levels. Cells were washed carefully, and filter inserts (with or without cells) moved to a 12-well plate (blocked overnight with PBS containing 1 % FCS), containing 950 µL pre-warmed media. Inserts were incubated apically with brain vehicles and integrity marker dex-TexR (ThermoFisher, D3328; 3000 Da) (0.5 µM of vehicle or 5 µM dex-TexR/well; total volume of 475 µL) at 37 °C for the duration of the experiment. Aliquots (80 µL) were collected from the basolateral chamber at various time points, and placed directly in a black 96-well plate (blocked o/n with PBS containing 1 % FCS) for fluorescence to be quantified immediately after attaining the final sample. For each time point, 40 µL of media in the apical chamber was removed to correct for any concentration changes based on the sampling procedure. Data was generated from three inserts with and without culture for each fluorescent probe co-incubated with the integrity marker. When each assay was performed, a functional transcytosis control was run in parallel. Here three wells

were reserved for apical incubation with Tf-A647 (ThermoFisher, T23366) and dex-TexR (Thermofisher, D1830; 70 000 Da) (100 µg/mL of each compound/well; total volume of 475 µL).

*$P_{app}$ calculation*

For detection, fluorescence units were transformed in µM or µg/mL for the vehicle or Tf-A647 control analysis respectively, using a linear working range. All readings were corrected for background fluorescence.

Cleared volume was calculated by the following equation and plotted as a function of time (min): Cleared volume (µL) = $(([C]_B \times V_B)/[C]_A)$. Where: $[C]_B$ = concentration of fluorescent probe in the basolateral compartment; $V_B$ = volume in the basolateral chamber, and; $[C]_A$ = initial concentration of fluorescent probe in the apical compartment. The slope of the clearance curve was estimated by linear regression analysis and represents the PS (i.e. permeability × surface area) product. Apparent permeability ($P_{app}$) was calculated using the following equation: $P_{app}$ (cm/min) = PS product ($cm^3$/min)/surface area of insert (1.12 $cm^2$), within the linear working range for each compound screened.[2,3]

*$P_{app}$ correction calculation*

To correct for contributions to fluorescence by paracellular diffusion, the transport ratio (TR) was defined using a similar sized dex to the vehicle (3000 Da) and calculated by the following equation: ($P_{app}$ integrity marker with endothelial monolayer)/($P_{app}$ integrity marker filters only). To obtain the corrected endothelial permeability value, TR is multiplied by the $P_{app}$ for the fluorescent probe with filters only, providing a rate that represents the expected value for paracellular diffusion based on results from the integrity marker. Subtracting this from the $P_{app}$ of the fluorescent probe in culture provides a value corresponding to the probes transcytosis rate, excluding contributions from paracellular flux.

*Binding ELISA assay*

The procedure was performed at room temp on a shaking platform using PBS-Tween (0.05%) for the wash steps. Nickel coated clear 96-well plates (Pierce) was coated overnight with 1 µg/ml of the extracellular subunit of TfR recombinant human protein, N-His Tag (Life Technologies) in 0.5 M Carbonate buffer. The plates were washed (5 × 150 µL) and blocked for 1 h with PBS containing 1% BSA. 100 µL of the compound dilutions in PBS were transferred to the plate and incubated for 1 h at room temp, after which time the plates were washed (5 × 150 µL). A Goat anti-FAM peroxidase labelled antibody (Abcam, 1:100 000) was added to the wells and incubated for 30 min. Following 6 washes, 50 µL of TMB Substrate Solution (Sigma) was added per well and incubated for a further 30 min. After addition of 50 µL of 2 M sulphuric acid to each well, receptor complexes were detected by measuring absorption at 450 nm. The procedure for determining the binding affinity of biotin labelled Tf (Sigma) was similar except streptavidin peroxidase (ThermoFisher Scientific) (1:20 000) was used to measure the Tf-receptor complex.

**Imaging and Immunochemistry**

*Colocalisation of delivery scaffolds with LAMP1 and Tf-A647*

bEND.3 cells were seeded at a density of 1 x $10^4$ cells/well in 8-well glass plates (ibidi) coated with rat tail collagen. After 3 days at 100 % confluence, cells were washed with PBS (3 × 180 µL), and co-incubated with brain delivery scaffolds (5 µM) and Tf-A647 (50 µg/ml) at 37 °C for 2 h in FluoroBrite DMEM media (Thermofisher). Cells were washed with PBS (3 × 180 µL), then fixed with 4 % paraformaldehyde for 15 min at room temperature. Cells were permeabilised with 0.1 % Triton X-100 for 20 min then blocked for 1 h to prevent non-specific binding using 5 % BSA with 0.1 % Tween. Cells were incubated at room temperature with primary antibody (Rabbit anti-LAMP1, Sigma-Aldrich; 1:100) for 1 h then rinsed with PBS (3

× 180 µL). Samples were protected from light and incubated for 1 h with secondary antibody (Goat anti-rabbit Rhodamine Red conjugate, Life Technologies; 1:500). Unbound antibody was removed through washing with PBS (5 × 180 µL). To stain the nuclei, cells were incubated with DAPI (1 µg/mL in PBS) for 15 min, washed with PBS (3 × 180 µL) then stored in mounting medium (150 µL; Citiflour™ glycerol PBS Solution AF1, Agar Scientific) before image visualisation.

*Imaging and BioImage Analysis*

The following channels were acquired: Nucleus (stained with DAPI, ex. 395/25 nm, em. 450/40 nm), scaffold (FAM-conjugated, ex. 470/24 nm, em. 525/50), LAMP1 (Alexa-Fluor 568, ex. 575/25 nm, em. 624/40) and Tf-A647 (Alexa-Fluor 647, ex. 640/30 nm, em. 690/50 nm). For z-stacks, frames of 2048 x 2048 pixels were acquired without binning at a pixel size of 0.11 µm with a z spacing of 0.24 µm. To perform three-dimensional channel alignment, images of 100 nm fluorescent beads (Zeiss) were acquired with settings identical to those used for cellular analysis. This was done for each experiment to account for possible variations in the hardware.

Channel alignment on beads was done in ZEN, parameters were saved and applied to all images from that experiment. Empty z slices after channel alignment were removed, and the histograms of LAMP1 and Transferrin were equalized in FIJI.[4]

Aligned images were deconvolved using Huygens (Scientific Volume Imaging b.v., The Netherlands) using a point spread function (PSF) which was distilled from the bead image for each experiment.

Three-dimensional colocalisation analysis was performed with Huygens, using the Object Analyser module. Briefly, all channels were segmented to create 3D objects. Segmented objects from the delivery vehicle channel were analysed for their 3D colocalisation with any

other objects from the DAPI, LAMP1 and Tf-A647 channels, to identify any voxel of the vehicle object that localised in space with a voxel from any object from the other three channels. The relative amount of voxels from each compound objects that colocalised (% colocalisation) were then calculated.

Data shown are mean percentage of colocalisation +/- 95% confidence interval of the mean of all objects found in three fields of view per scaffold, corresponding to a range of 125-219 cells, depending on the delivery vehicle. Total number of objects analysed are as follows: vehicle (1342-13244), nuclei (125-219), LAMP1 (11108-40054) and Tf-A647 (35021-51445).

Micrographs were prepared with Icy (http://icy.bioimageanalysis.org),[5] and Huygens.

## Part 2. Supporting Figures

### Figure S1

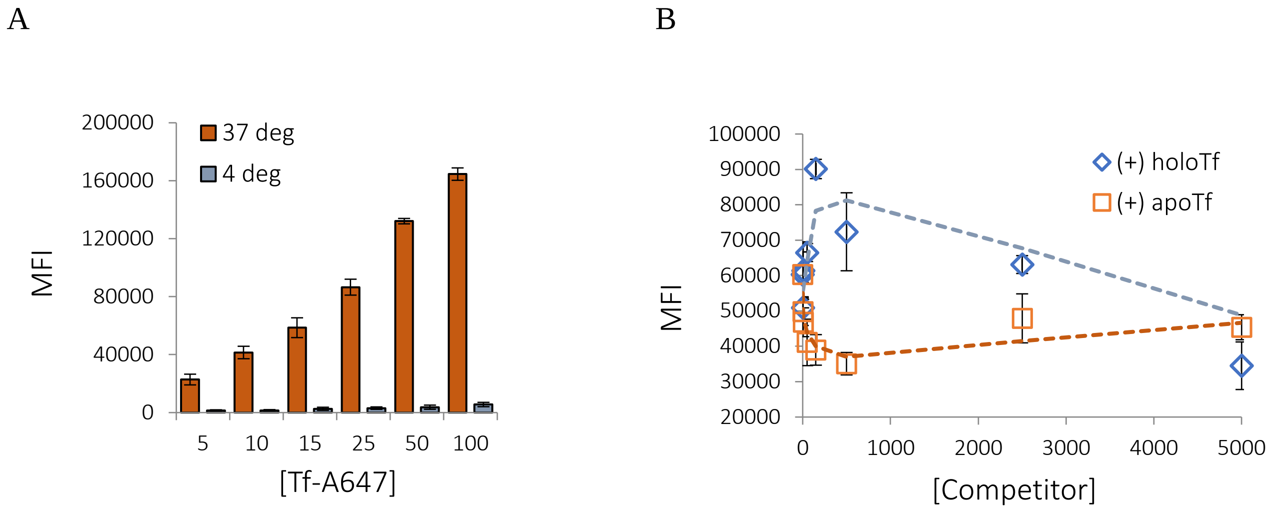


**Figure S1 FC data obtained with Tf-A647 in bEND.3 cells.** (A) Dose-response uptake of Tf-A647 at physiological temperature (red) which is inhibited at 4 °C (blue); (B) Average mean fluorescence intensity (MFI) uptake of Tf-A647 (10 µg/ml) when co-incubated with either *holo-* or *apo*-Tf over different concentrations.

### Figure S2

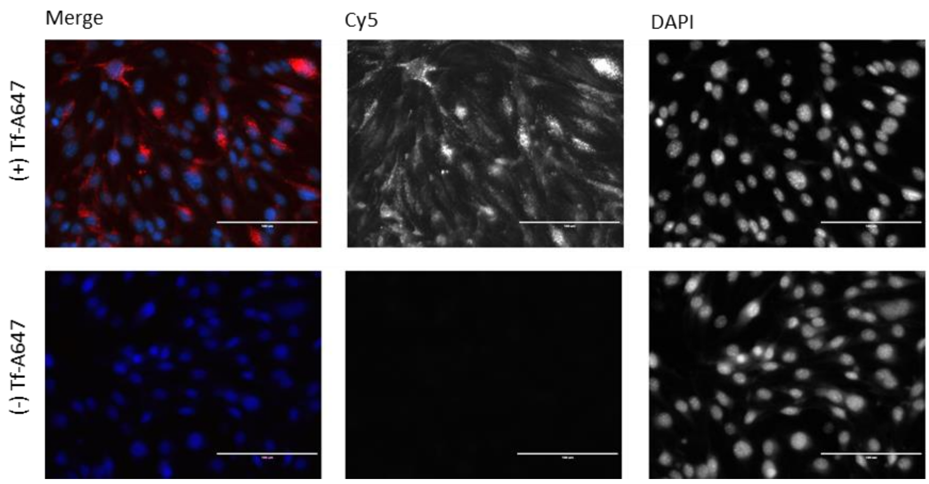


**Figure S2 Wild-field (WF) images of Tf-A647 (25 µg/ml) endocytosis following 3 h incubation at 37 °C in bEND.3 cells.** bEND.3 cells were seeded at a density of 1 x $10^4$ cells/well 8-well plates and grown for 6 days prior the experiment. Cells were incubated with DMEM containing Tf-A647 (180 µL per well) at 37 °C for 3 h then washed (3 × 180 µL PBS) and fixed with 4 % paraformaldehyde for 15 min at room temperature. To stain the nuclei, cells were incubated with DAPI (1 µg/mL in PBS) for 15 min, washed with PBS (3 × 180 µL) then stored in PBS (150 µL) in the dark until image acquisition. Scale bar represents 100 µm.

Figure S3

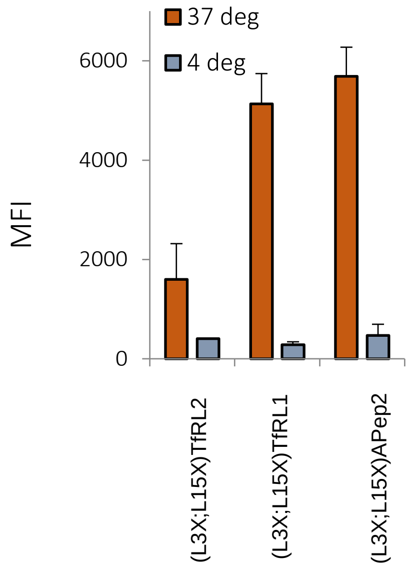


**Figure S3 Temperature dependence for uptake of bivalent scaffolds (0.5 µM) in media by FC.** Vehicles were continuously fed to bEND.3 cells for 3 h, stained with DAPI, washed, extracellular fluorescence quenched with trypan blue and measured for fluorescent signal. MFI = mean fluorescent intensity.

Figure S4

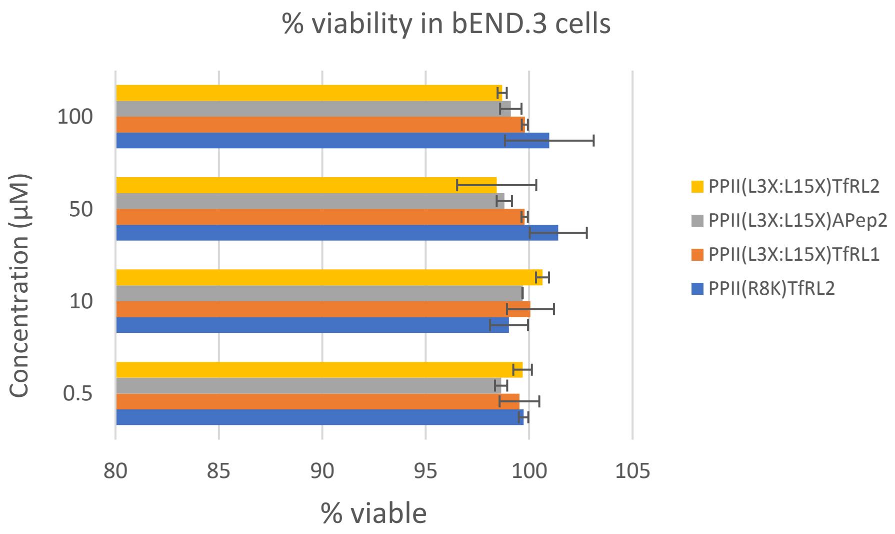


**Figure S3 Cytotoxicity of selection of vehicles over a concentration range by FC.** Vehicles were continuously fed to bEND.3 cells for 3 h, stained with DAPI, washed, and measured for fluorescent signal. Percentages were obtained by comparison to cells treated in an equivalent fashion without the addition of vehicle.

Figure S5

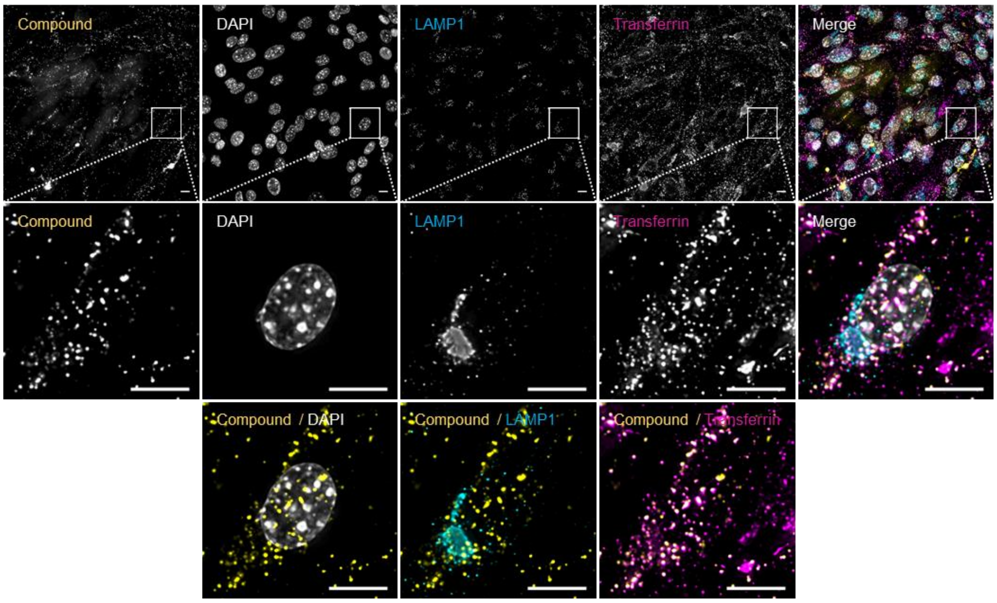


**Figure S5 Imaging of BBB vehicles in bEND.3 cells with Tf-A647, LAMP1 and DAPI for quantification of colocalisation.** Cells are immunostained for LAMP1 (cyan), counterstained with DAPI (grey), and incubated with a BBB vehicle (yellow; PPII(L3X;V13X)TfRL1) and Tf-A647 (magenta). Images shown are maximum intensity projections of the deconvolved z-stack. Top row shows a whole field of view, middle shows a single cell and bottom shows overlays of compound with DAPI, LAMP1 and Tf-A647. 3D signal was used to create 3D objects in all channels (shown in Fig 3 of manuscript) and the percentage of overlapping voxels between each object was calculated to assess colocalisation of the vehicle with LAMP1 and Tf-A647. Scale bar represents 10 µm.

Figure S6

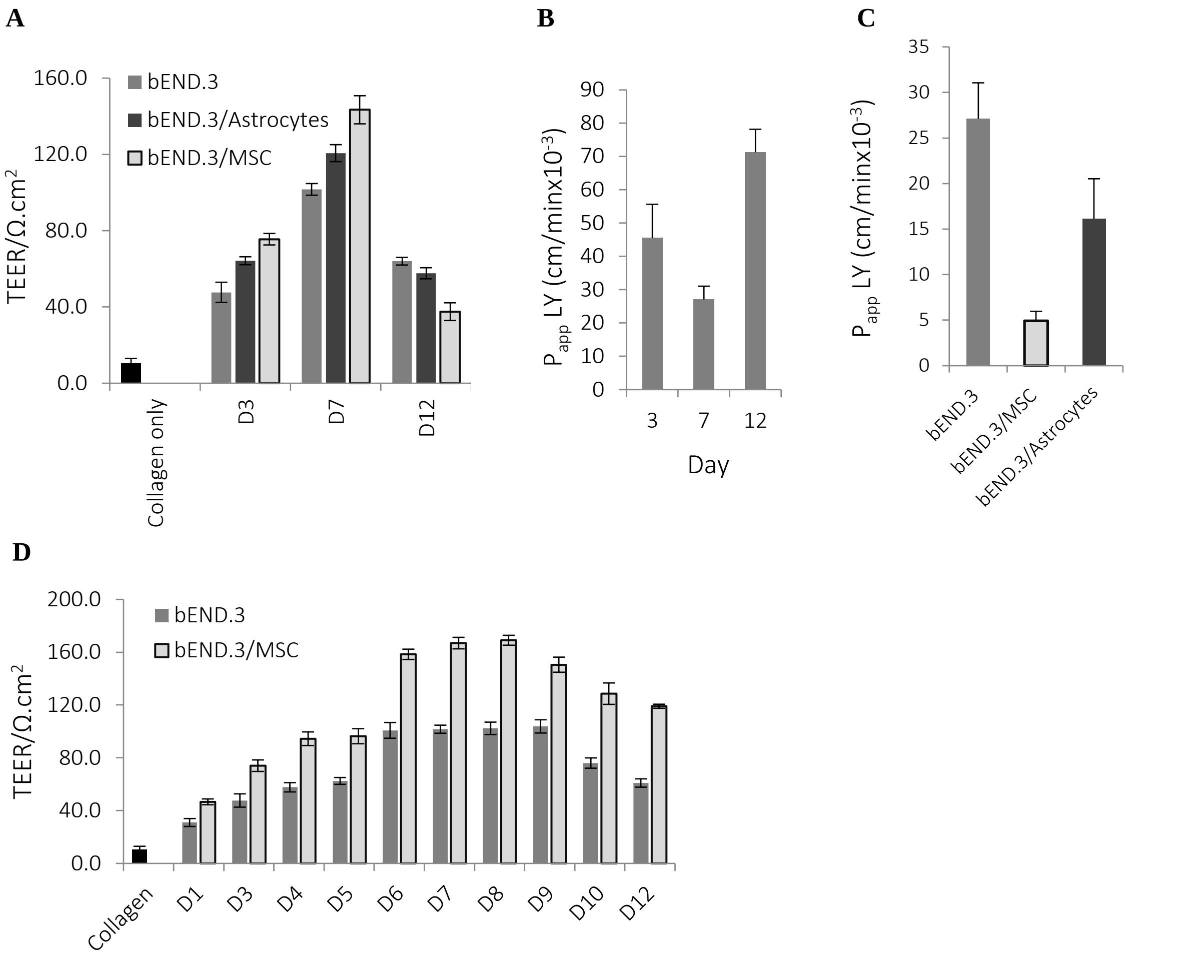


**Figure S6 Experimental TEER and $P_{app}$ values for paracellular diffusion marker LY in various *in vitro* BBB models.** (A) TEER for bEND.3 or bEND.3 co-culture system at defined days. For all TEER measurements, media was changed 24 h prior to each reading. (B) Experimental $P_{app}$ values for paracellular diffusion marker LY for bEND.3 monolayer over time. (C) Experimental $P_{app}$ values for paracellular diffusion marker LY in co-culture at optimal times for seeding. (D) Experimental TEER for bEND.3 or bEND.3/MSC co-culture system. Each measurement represents a replicate of n=5. Key: D = day.

Figure S7

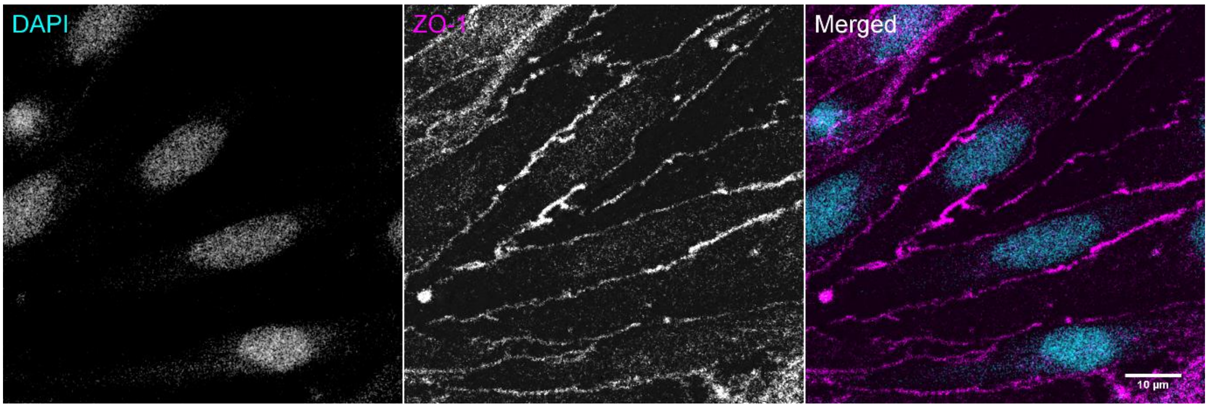


**Figure S7 Immunofluorescent staining of confluent bEND.3 cells for TJ protein ZO-1 (red) and nucleus (blue) by confocal microscopy.** A continuous and smooth staining pattern for TJ markers at cell junctions is shown. bEND.3 cells were seeded at a density of 1 x $10^4$ cells/well on 8-well collagen IV coated After 3 days at 100 % confluence, cells were washed with PBS, fixed with 4 % paraformaldehyde for 15 min at room temperature, then permeabilised and blocked to prevent non-specific binding using 5 % BSA with 0.1 % Triton X-100. Cells were incubated at room temperature with primary antibody (Rabbit anti-ZO1, Life Technologies; 1:100) for 1 h then rinsed with PBS. Samples were protected from light and incubated for 1 h with secondary antibody (Donkey anti-rabbit Alexa Fluor®555 conjugate, Abcam; 1:500). Unbound antibody was removed through washing with PBS (5 × 180 µL). To stain the nuclei, cells were incubated with DAPI (1 µg/mL in PBS) for 15 min, washed with PBS (3 × 180 µL) then stored in PBS (150 µL) before image visualisation.

Figure S8

A 1 2 3

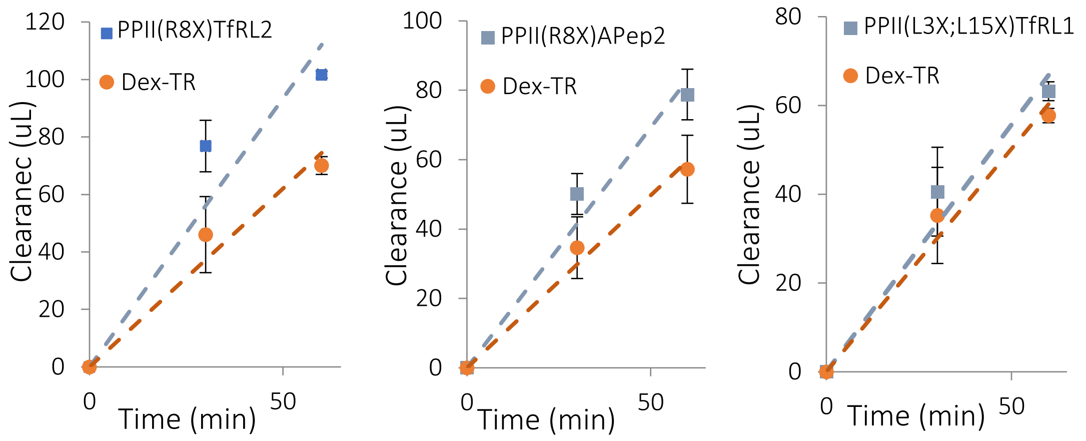


B 1 2 3

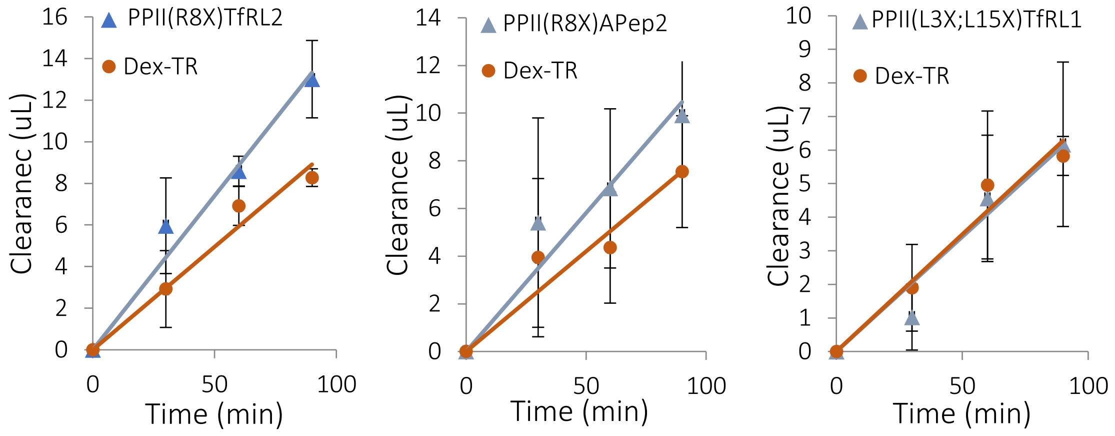


**Figure S8 Clearance plots for a selection of FAM-labelled vehicles at 4 °C.** (1) and (2) represent monovalent vehicles fused to TfRL2 or APep2 respectively, while (3) shows the passage of a TfRL1 bivalent vehicle without (A; dashed lines) and with cells (B; solid lines). $_{c}P_{app}$ values were determined as -0.085 cm/min x $10^{-5}$, -0.058 cm/min x $10^{-5}$ and -0.079 cm/min x $10^{-5}$ respectively. $_{c}P_{app}$ for Tf-A647 at 4 deg was 0.09 cm/min x $10^{-5}$ (data not shown).

## Part 3. Characterisation data for BBB shuttles

3.1 UV and Mass spectrometry data for SAP and peptide ligands

*FAM-SAP*

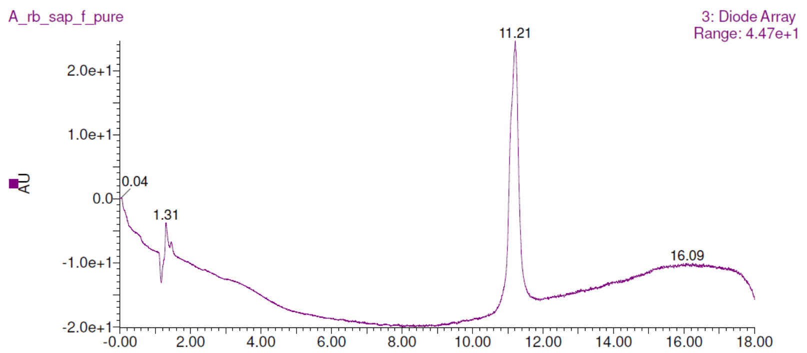


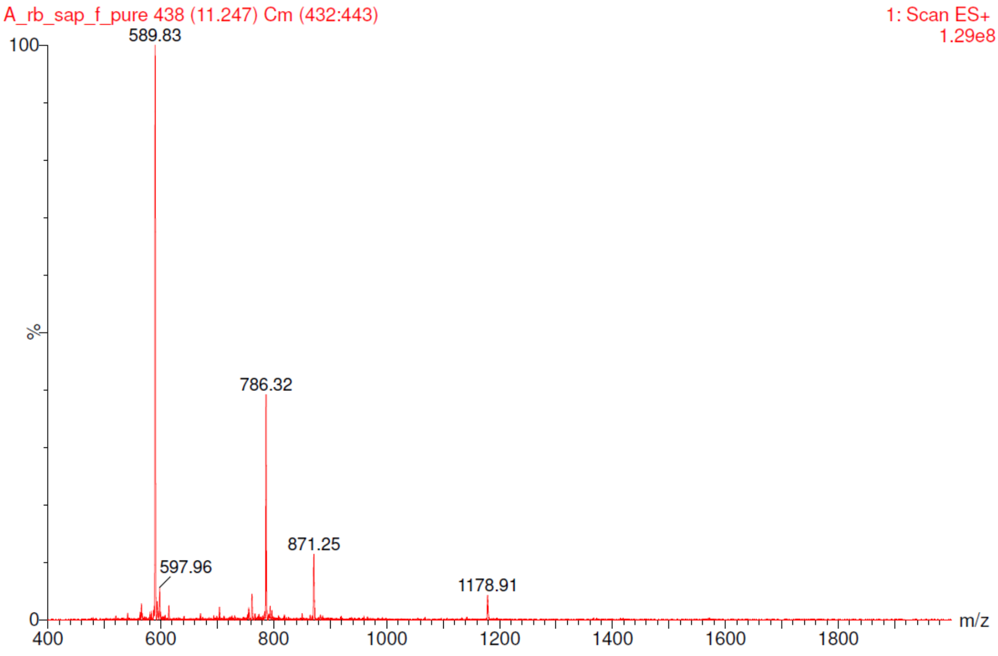

*FAM-TFRL1*

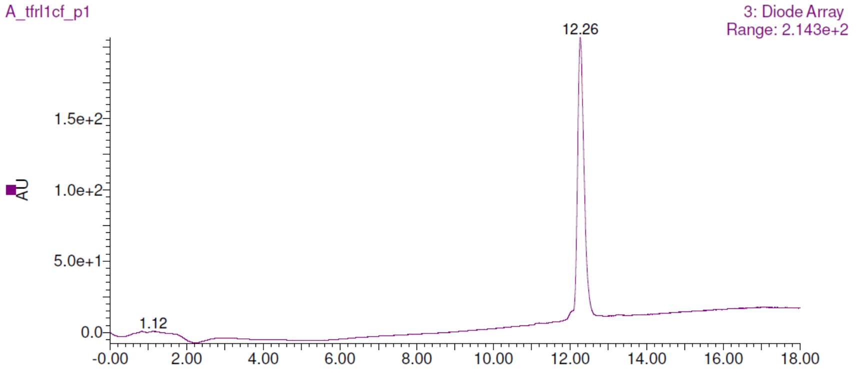

A_tfrl1cf_p1
3: Diode Array
Range: 2.143e+2
12.26
1.5e+2
1.0e+2
AU
5.0e+1
0.0
1.12
-0.00
2.00
4.00
6.00
8.00
10.00
12.00
14.00
16.00
18.00


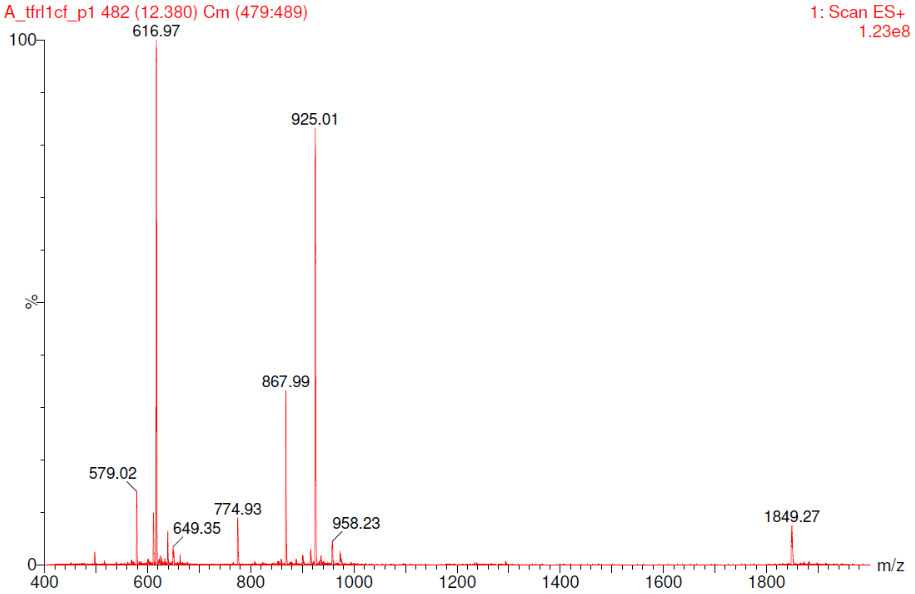

A_tfrl1cf_p1 482 (12.380) Cm (479:489)
1: Scan ES+
1.23e8
100
616.97
925.01
%
867.99
579.02
774.93
649.35
958.23
1849.27
0
m/z
400
600
800
1000
1200
1400
1600
1800

*FAM-TFRL2*

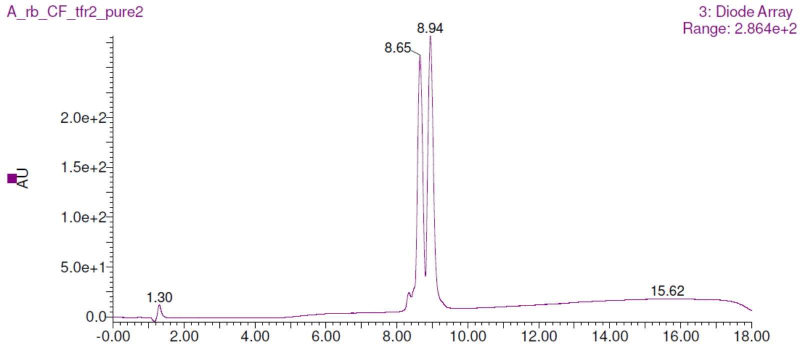

A_rb_CF_tfr2_pure2
3: Diode Array
Range: 2.864e+2
8.94
8.65
2.0e+2
1.5e+2
1.0e+2
5.0e+1
0.0
AU
1.30
15.62
-0.00
2.00
4.00
6.00
8.00
10.00
12.00
14.00
16.00
18.00


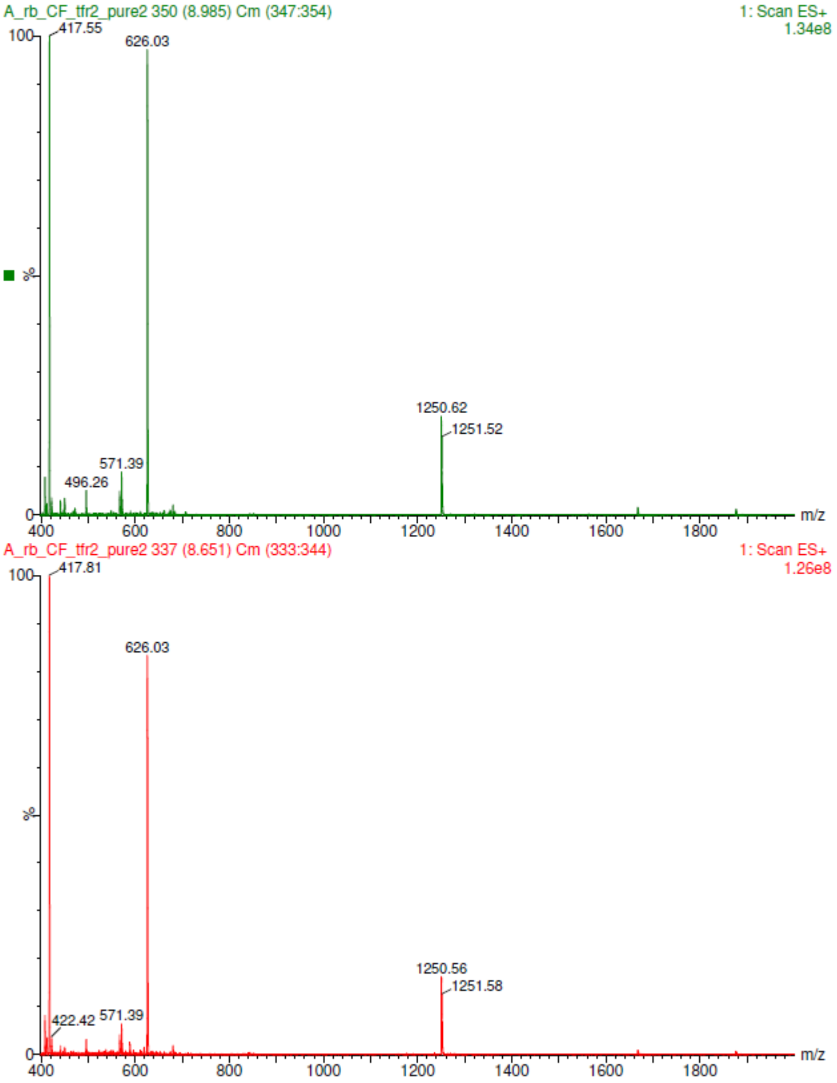

A_rb_CF_tfr2_pure2 350 (8.985) Cm (347:354)
1: Scan ES+
1.34e8
100
417.55
626.03
%
1250.62
1251.52
571.39
496.26
0
400
600
800
1000
1200
1400
1600
1800
m/z
A_rb_CF_tfr2_pure2 337 (8.651) Cm (333:344)
1: Scan ES+
1.26e8
100
417.81
626.03
%
1250.56
1251.58
422.42
571.39
0
400
600
800
1000
1200
1400
1600
1800
m/z

FAM-APep2

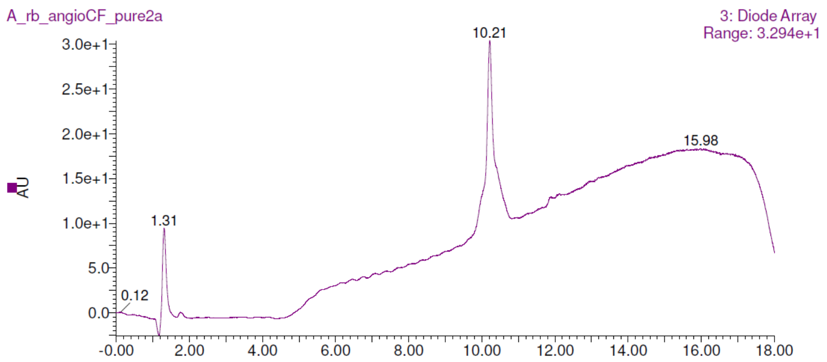
A_rb_angioCF_pure2a
3: Diode Array
Range: 3.294e+1
AU
10.21
15.98
1.31
0.12
3.0e+1
2.5e+1
2.0e+1
1.5e+1
1.0e+1
5.0
0.0
-0.00
2.00
4.00
6.00
8.00
10.00
12.00
14.00
16.00
18.00

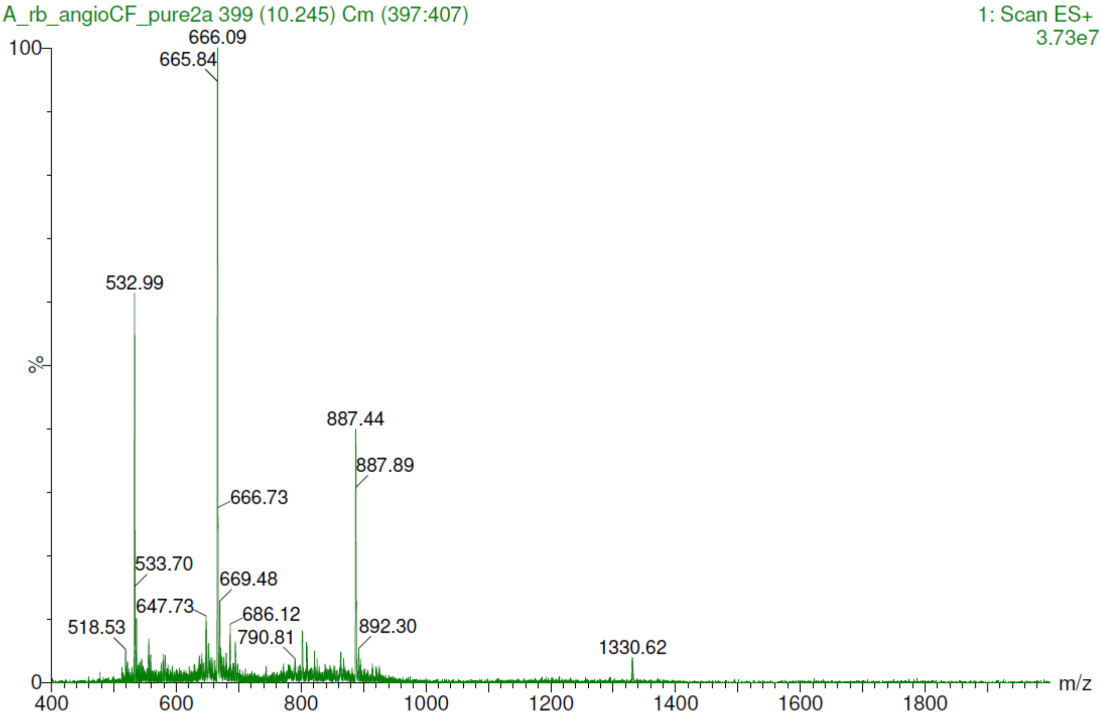
A_rb_angioCF_pure2a 399 (10.245) Cm (397:407)
1: Scan ES+
3.73e7
100
%
0
666.09
665.84
532.99
887.44
887.89
666.73
533.70
669.48
647.73
686.12
518.53
790.81
892.30
1330.62
m/z
400
600
800
1000
1200
1400
1600
1800

K($N_3$)TfRL1

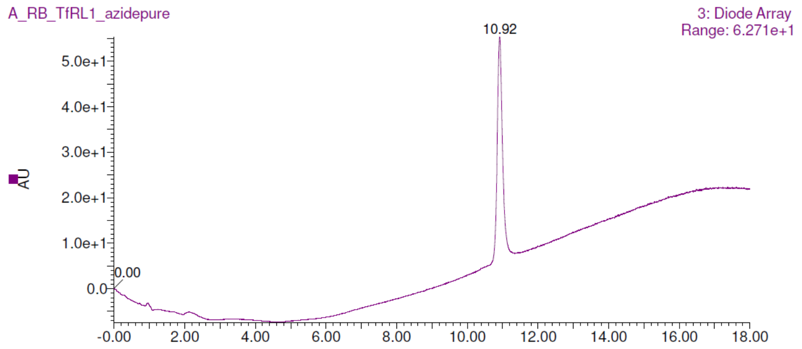

A_RB_TfRL1_azidepure
3: Diode Array
Range: 6.271e+1
10.92
5.0e+1
4.0e+1
3.0e+1
2.0e+1
1.0e+1
0.0
AU
0.00
-0.00
2.00
4.00
6.00
8.00
10.00
12.00
14.00
16.00
18.00


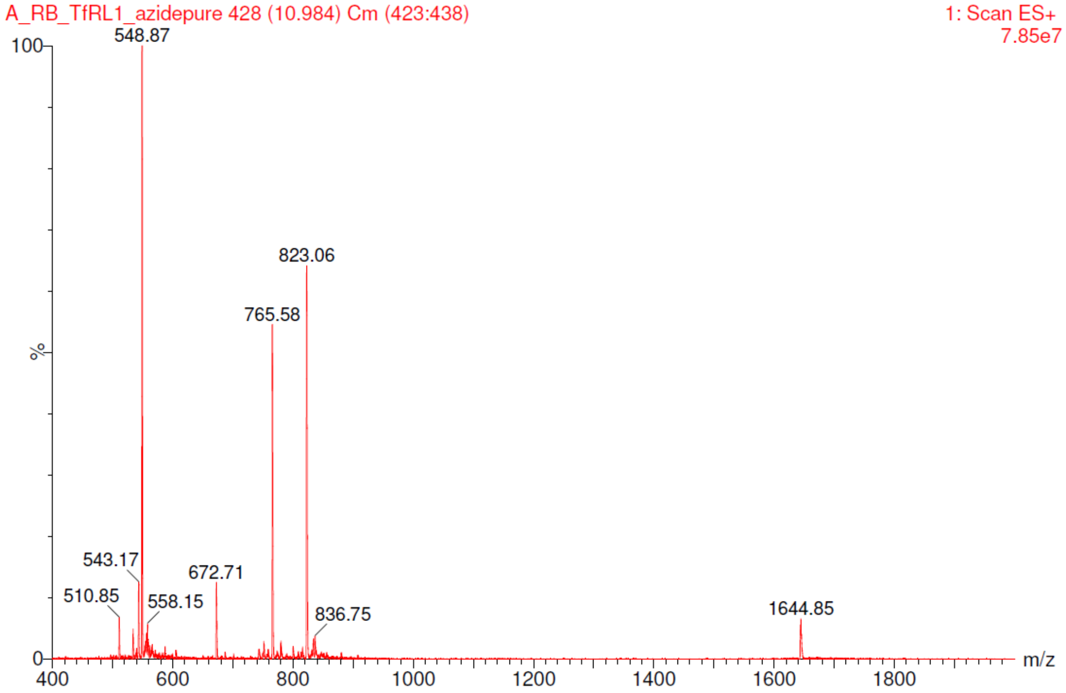

A_RB_TfRL1_azidepure 428 (10.984) Cm (423:438)
1: Scan ES+
7.85e7
548.87
823.06
765.58
543.17
672.71
510.85
558.15
836.75
1644.85
100
%
0
400
600
800
1000
1200
1400
1600
1800
m/z

K($N_3$)TfRL2

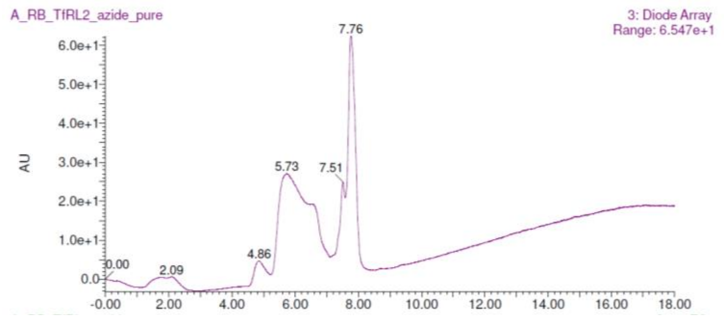

A_RB_TfRL2_azide_pure
3: Diode Array
Range: 6.547e+1
7.76
6.0e+1
5.0e+1
4.0e+1
3.0e+1
2.0e+1
1.0e+1
0.0
AU
5.73
7.51
4.86
0.00
2.09
-0.00
2.00
4.00
6.00
8.00
10.00
12.00
14.00
16.00
18.00


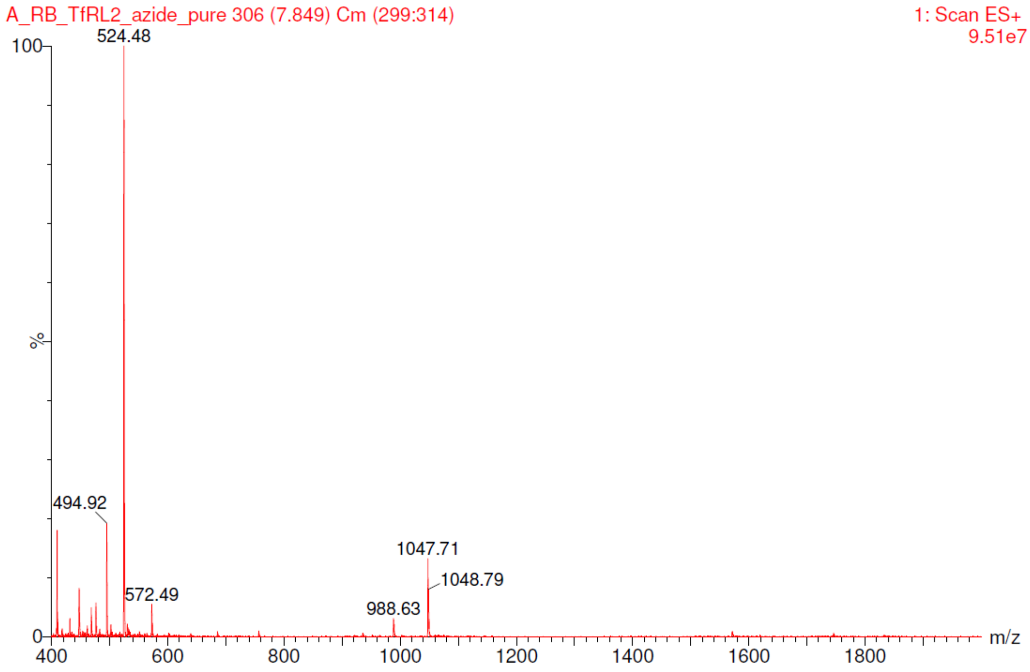

A_RB_TfRL2_azide_pure 306 (7.849) Cm (299:314)
1: Scan ES+
9.51e7
100
524.48
%
494.92
1047.71
1048.79
572.49
988.63
0
400
600
800
1000
1200
1400
1600
1800
m/z

K($N_3$)APep2

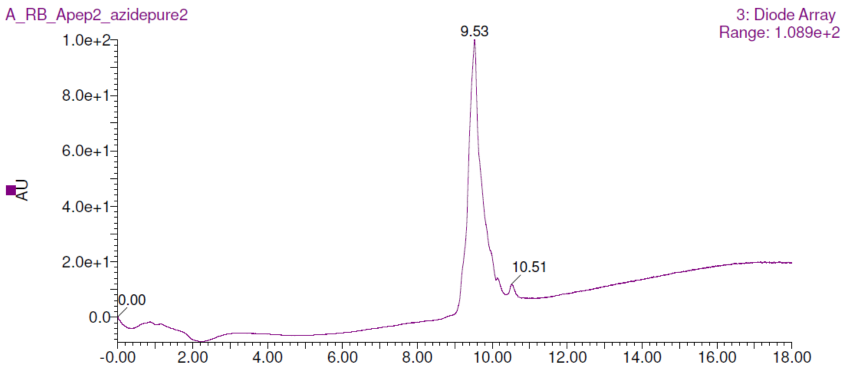

A_RB_Apep2_azidepure2
3: Diode Array
Range: 1.089e+2
AU
1.0e+2
8.0e+1
6.0e+1
4.0e+1
2.0e+1
0.0
9.53
10.51
0.00
-0.00
2.00
4.00
6.00
8.00
10.00
12.00
14.00
16.00
18.00


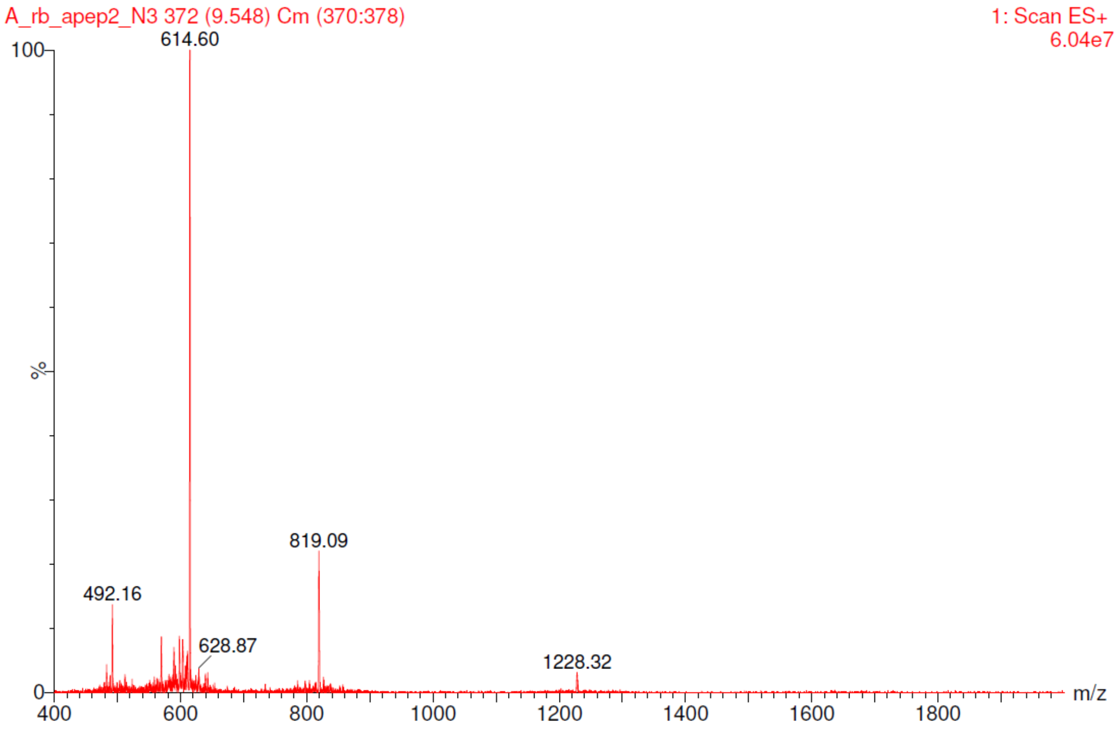

A_rb_apep2_N3 372 (9.548) Cm (370:378)
1: Scan ES+
6.04e7
100
%
0
614.60
819.09
492.16
628.87
1228.32
400
600
800
1000
1200
1400
1600
1800
m/z

3.1 Mass spectrometry data for BBB shuttles

*PPII(R8X)TfRL1*

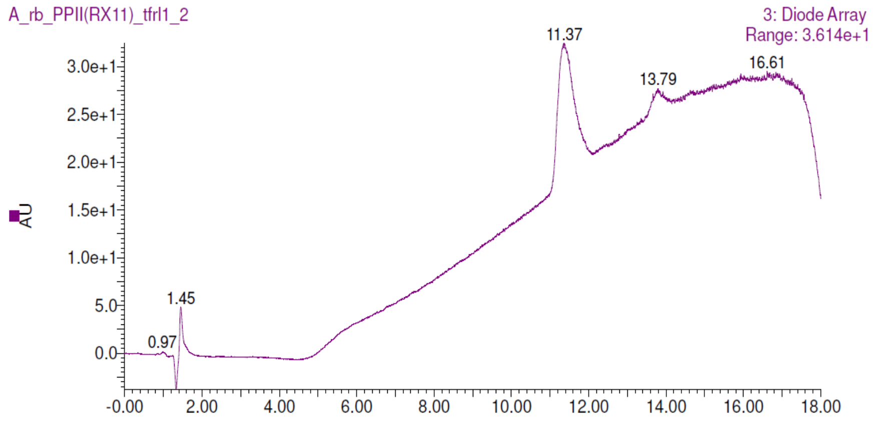


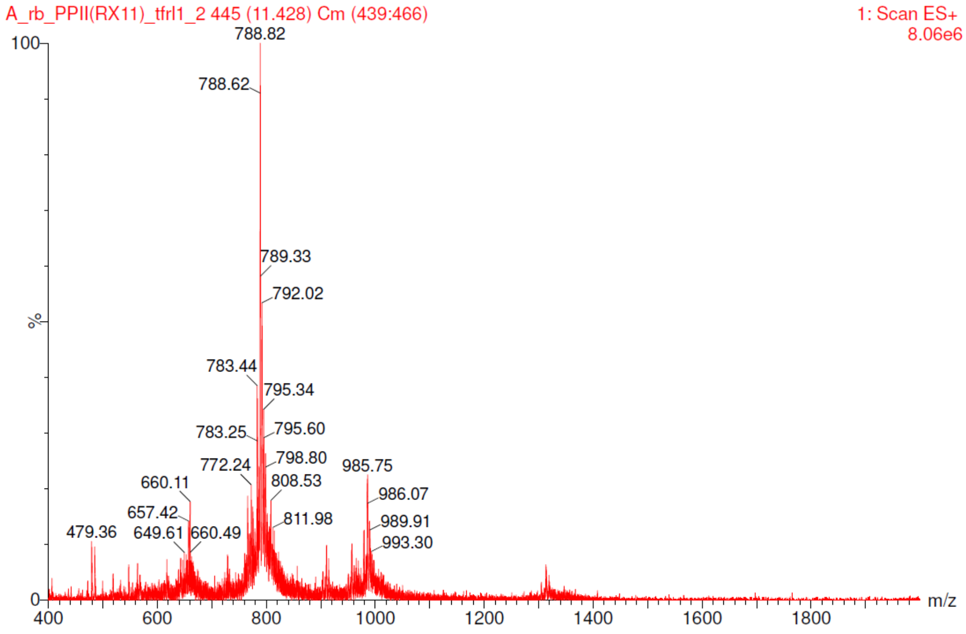

*PPII(R8X)TfRL2*

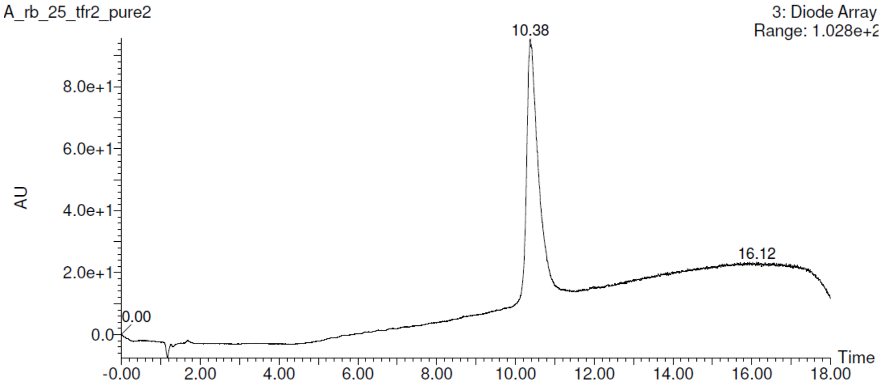


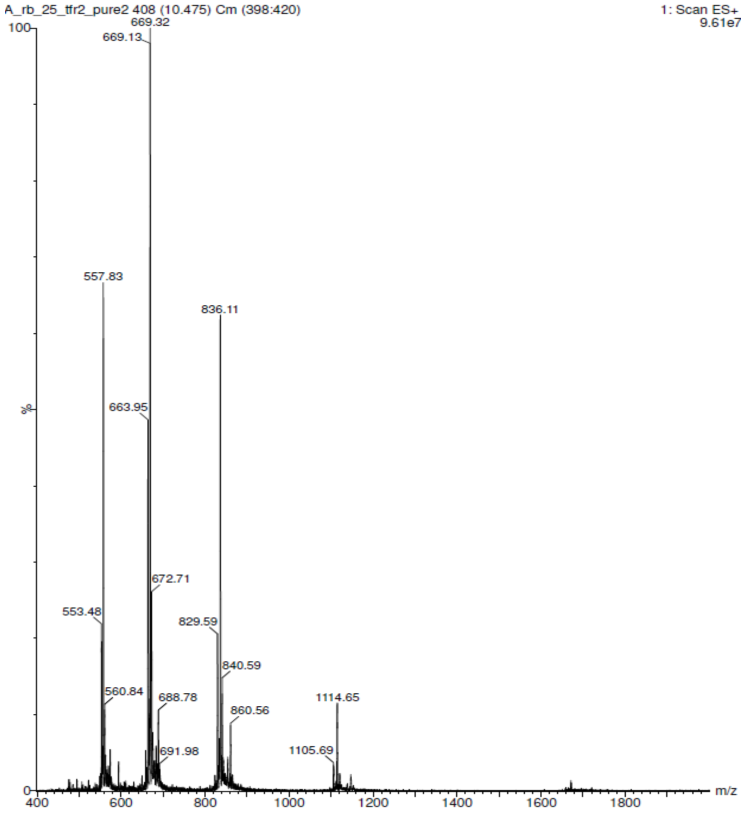

*PPII(R8X)APep2*

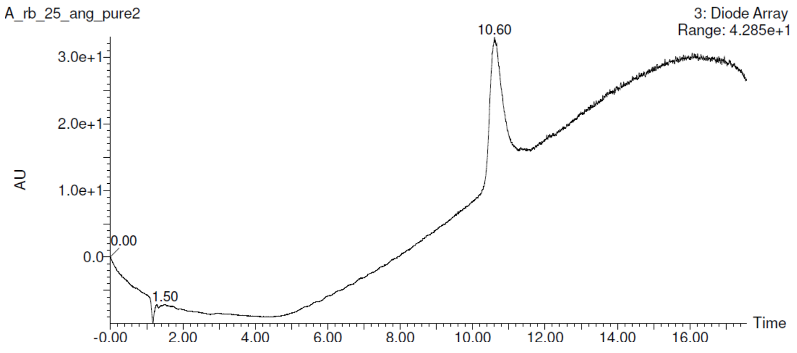


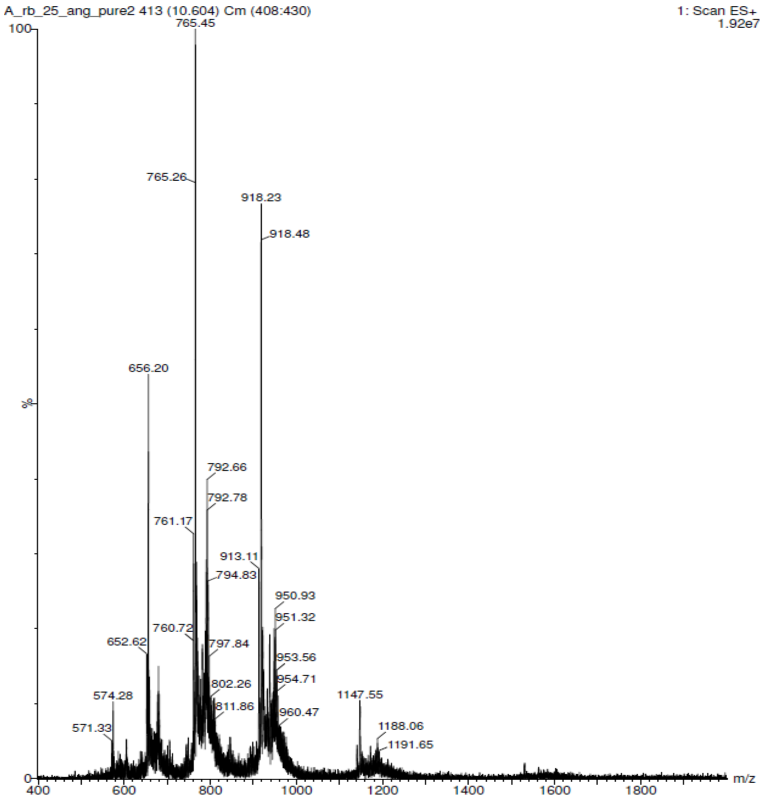

*PPII(R8K)TfRL2*

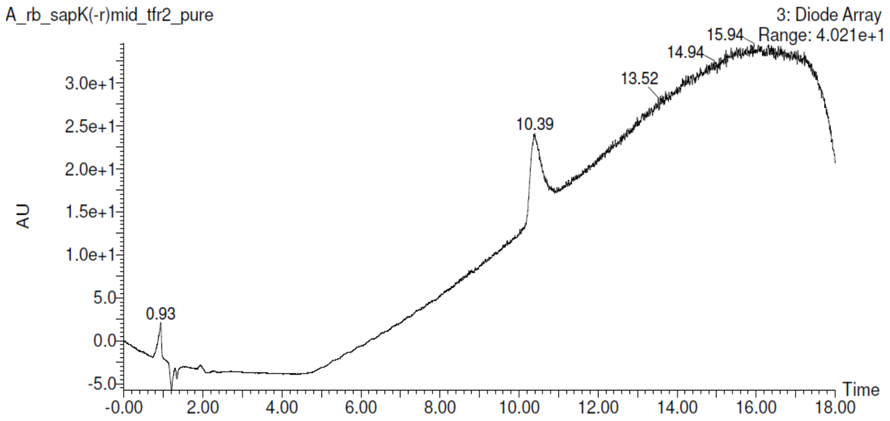


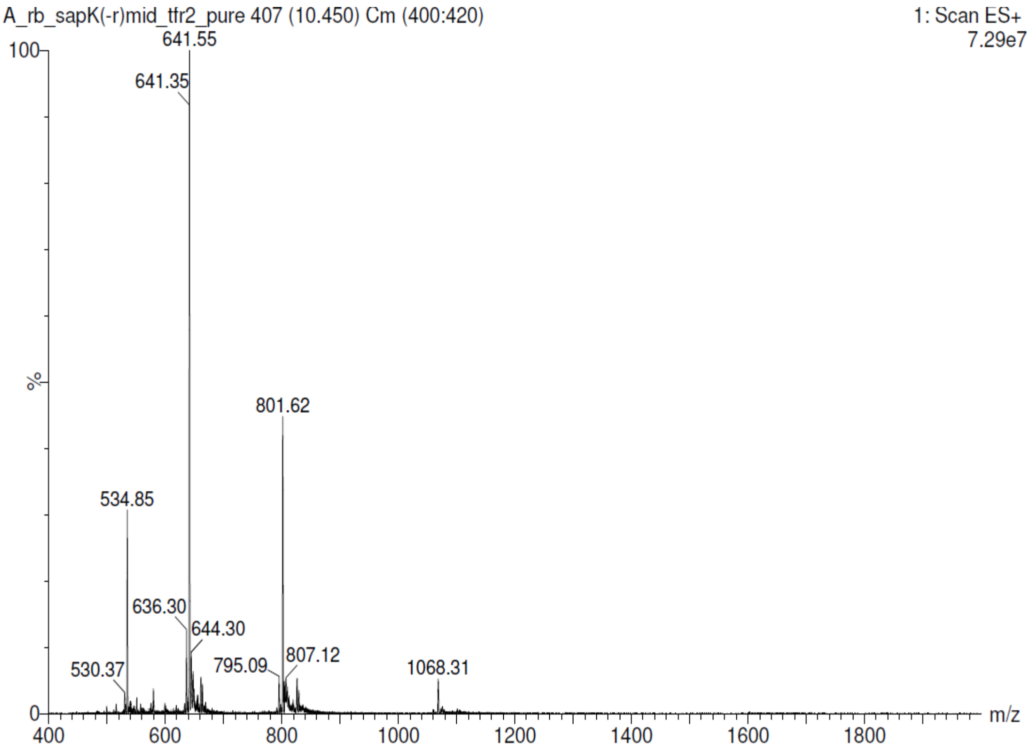

*PPII(L3X)TfRL2*

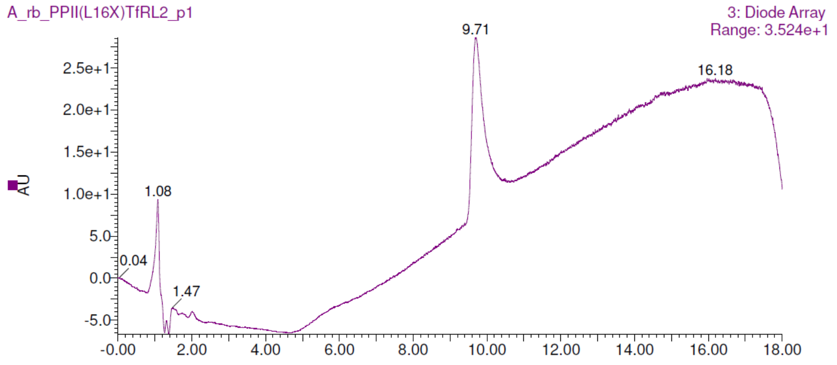


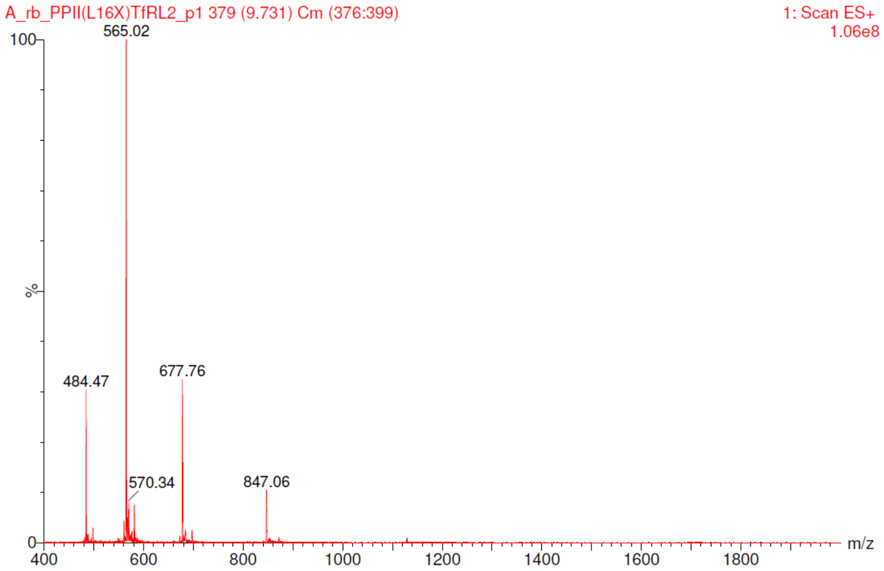

*PPII(LX15)TfRL2*

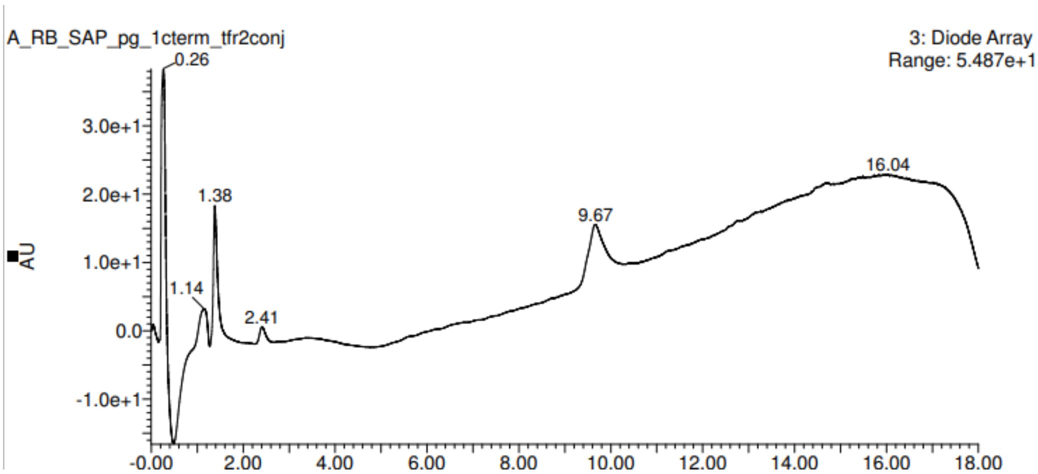


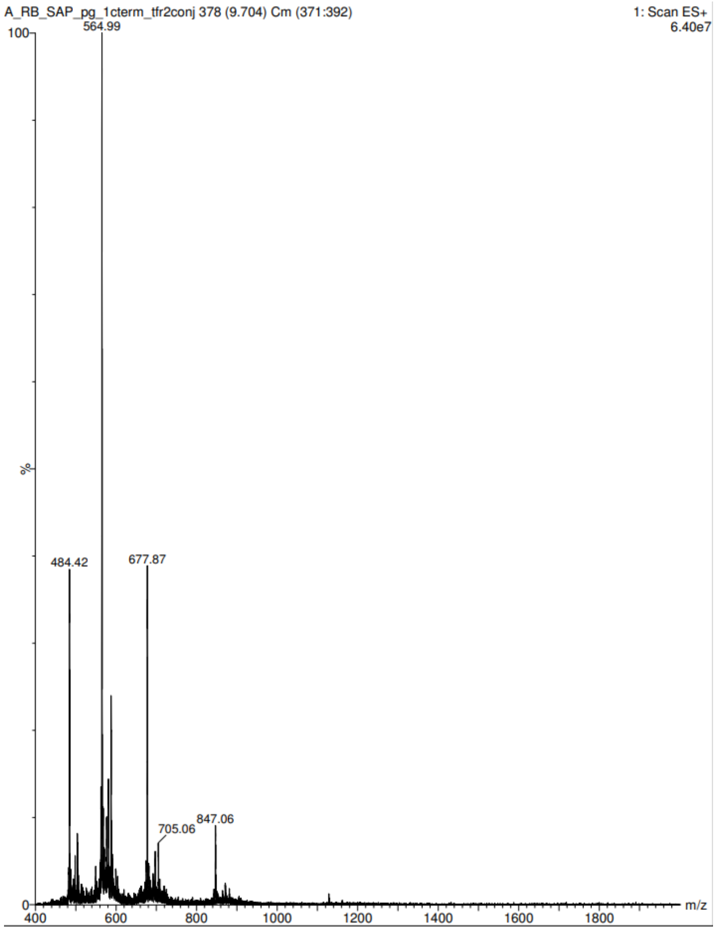

*PPII(L3X;L15X)TfRL1*

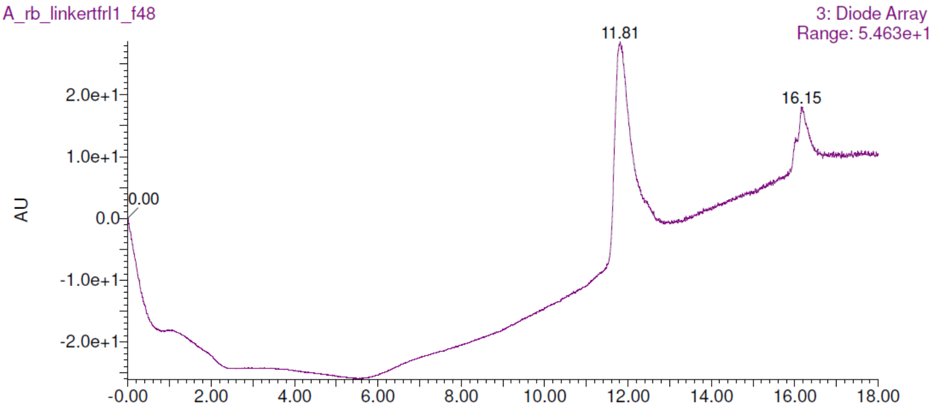


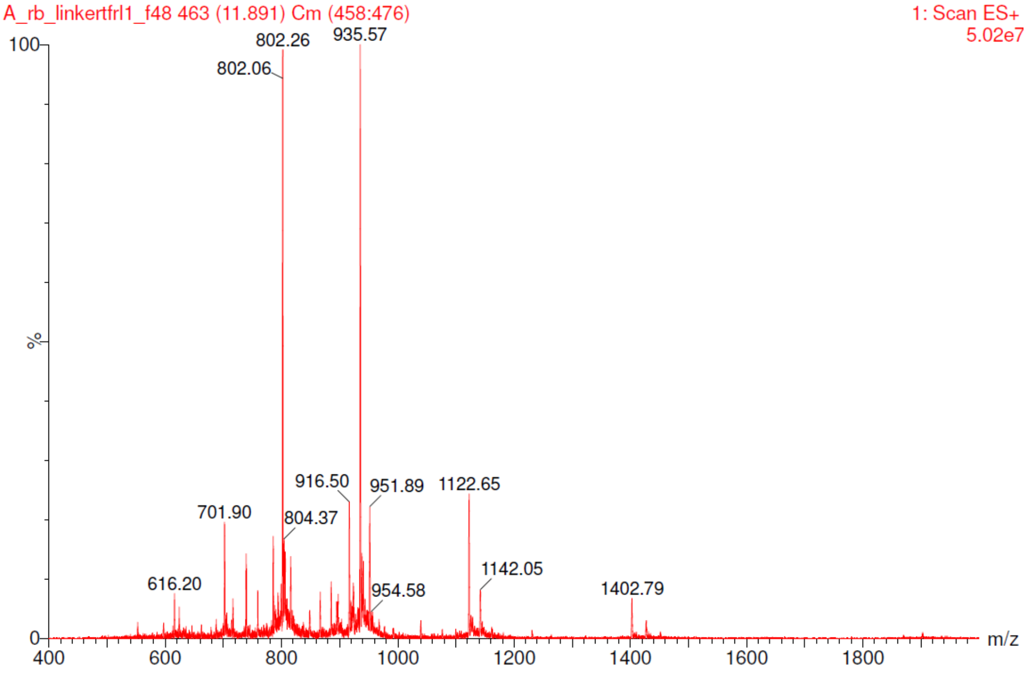

*PPII(L3X;L15X)TfRL2*

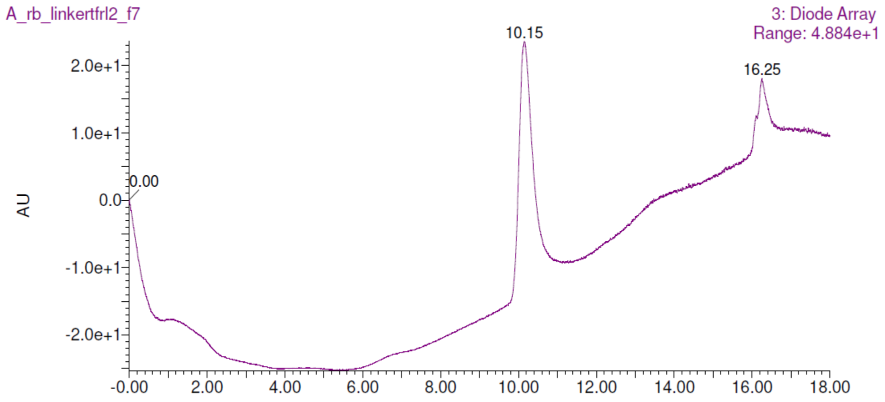


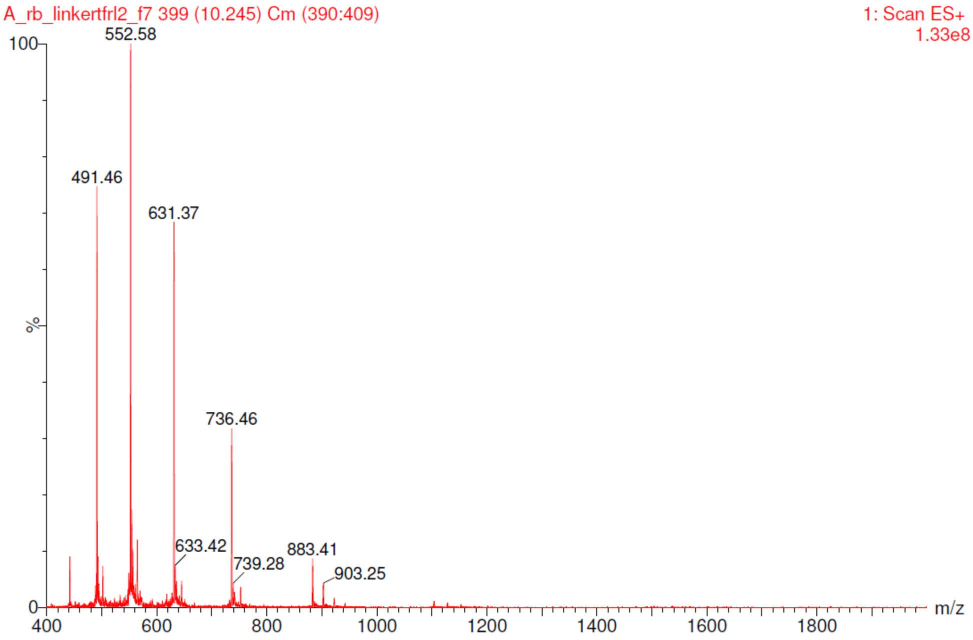

*PPII(L3X;L15X)APep2*

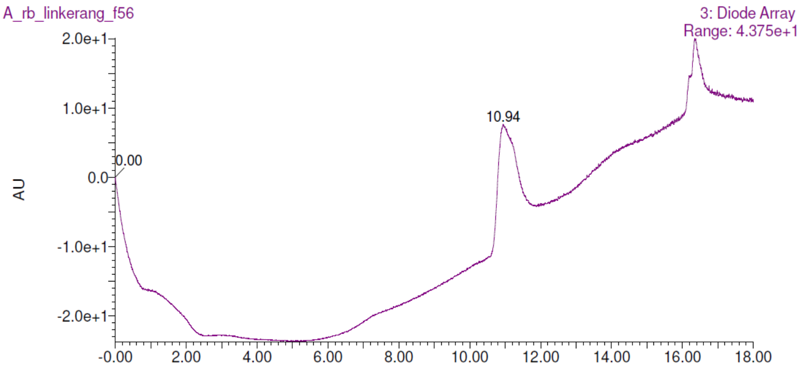


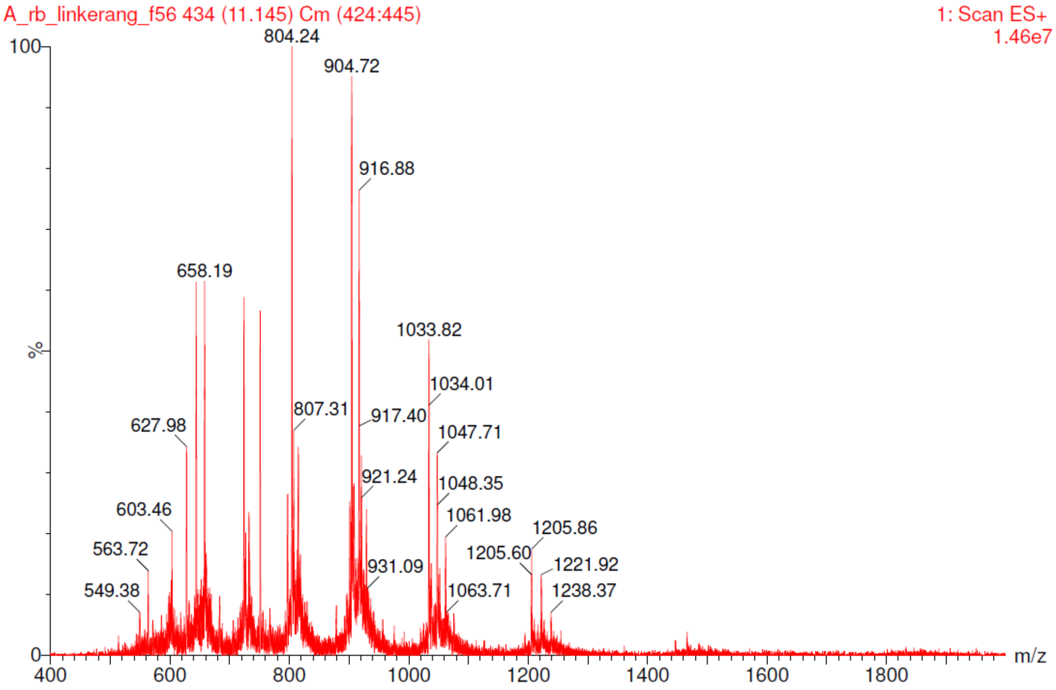

*PPII(L3X;V13X)TfRL1*

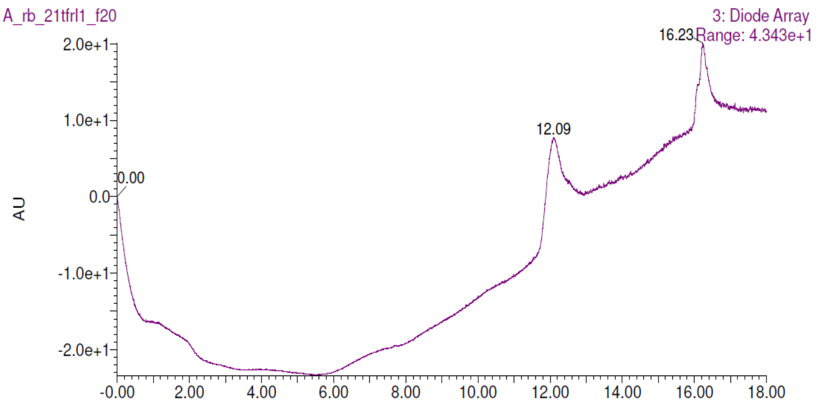


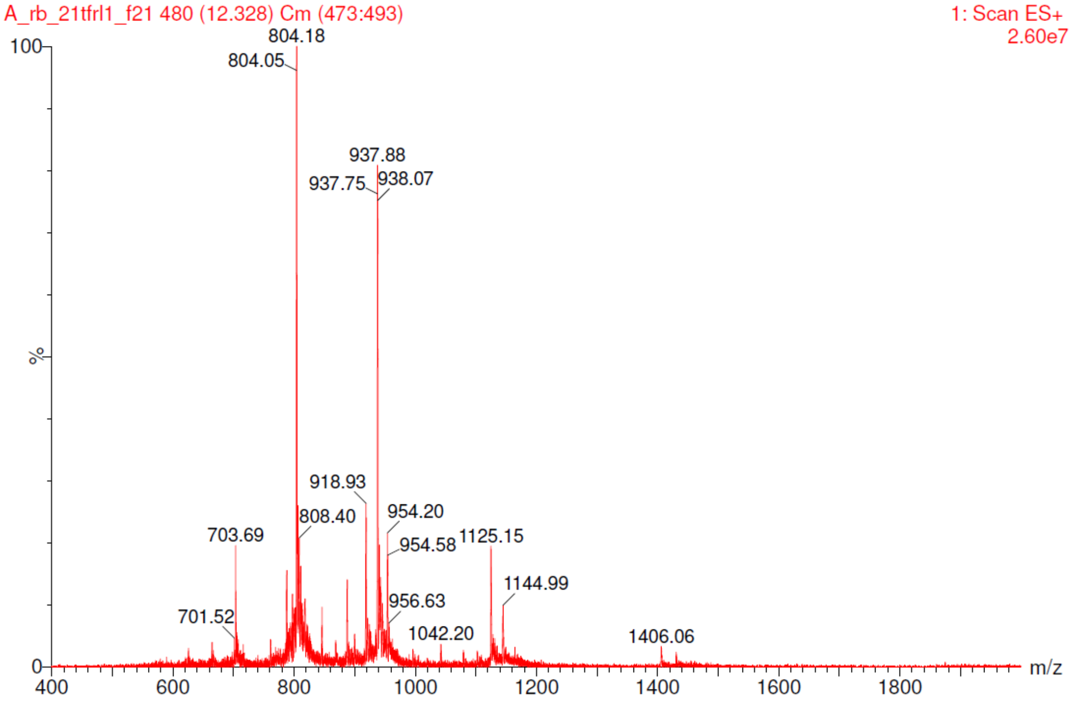

*PPII(L3X;V13X)TfR2*

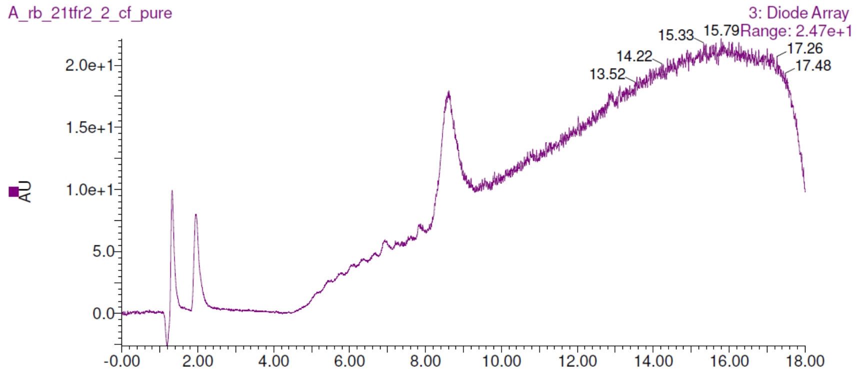


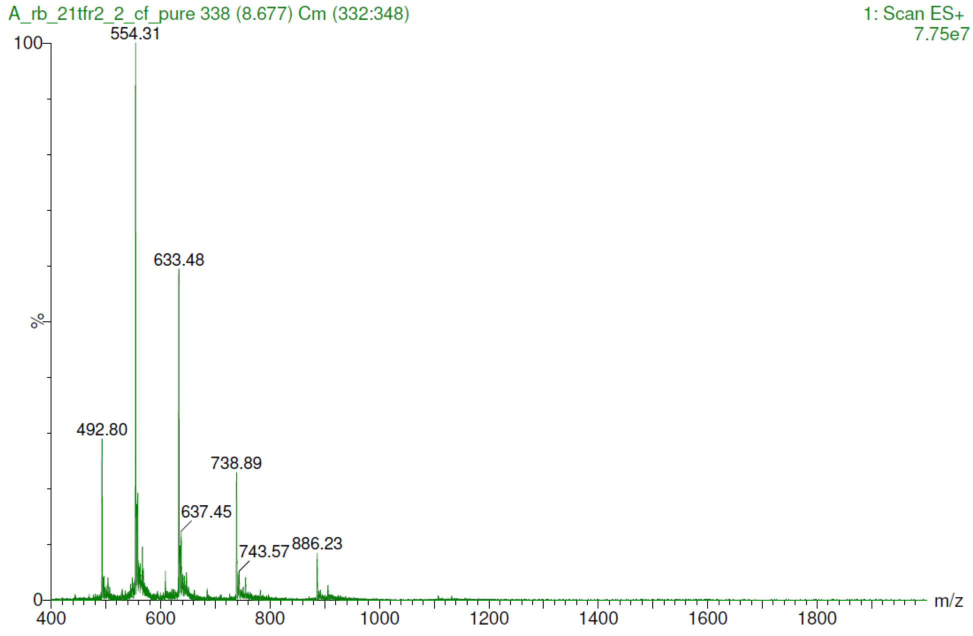

*PPII(L3X;V13X)APep2*

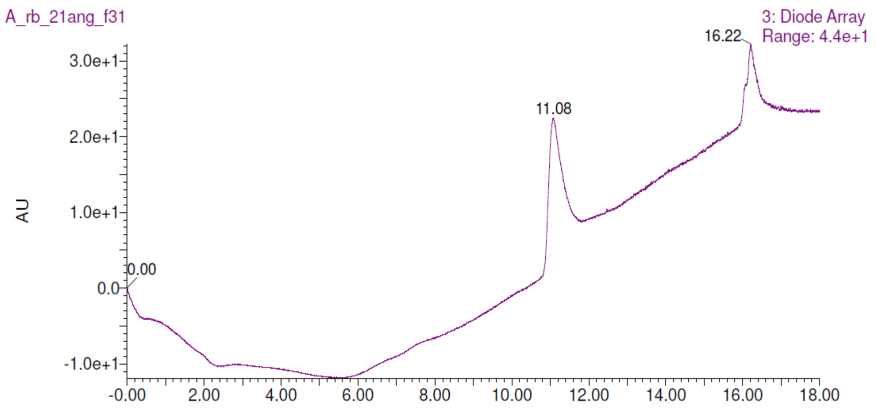


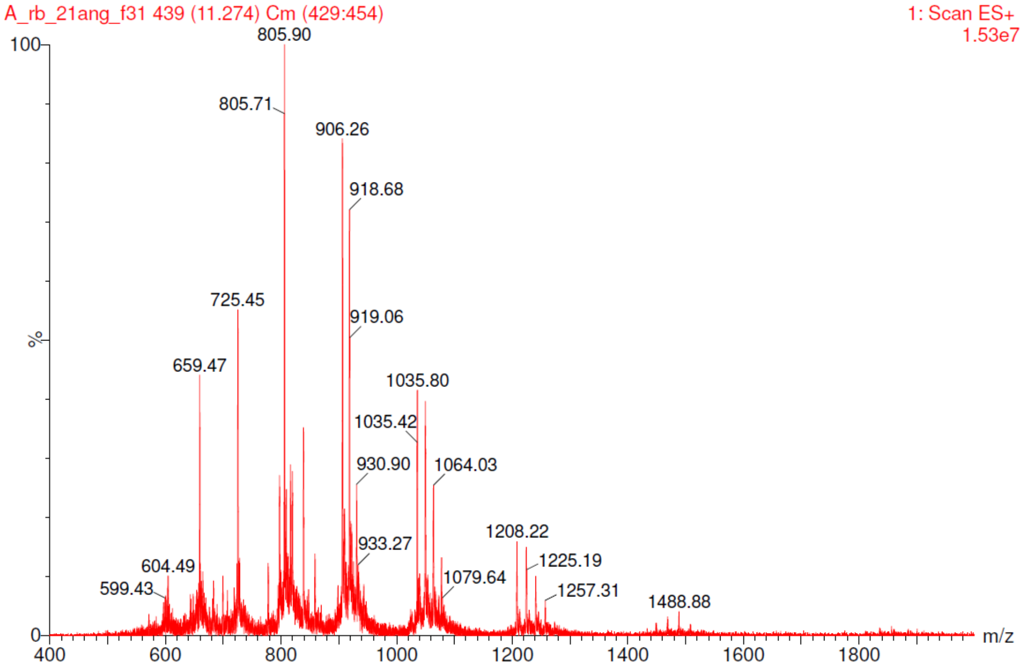

*PPII(L3X;L15K)TfRL2(K)TfRL1(X)*

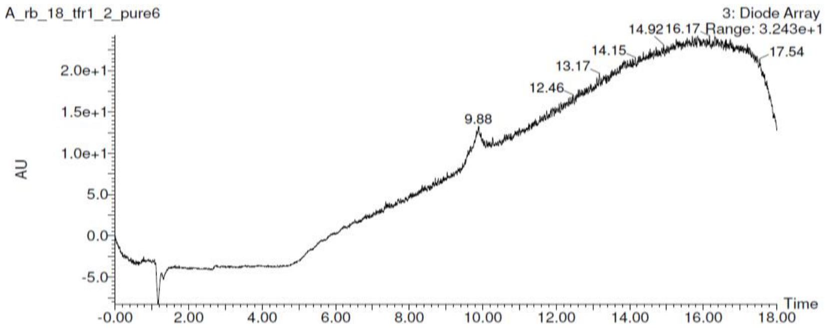


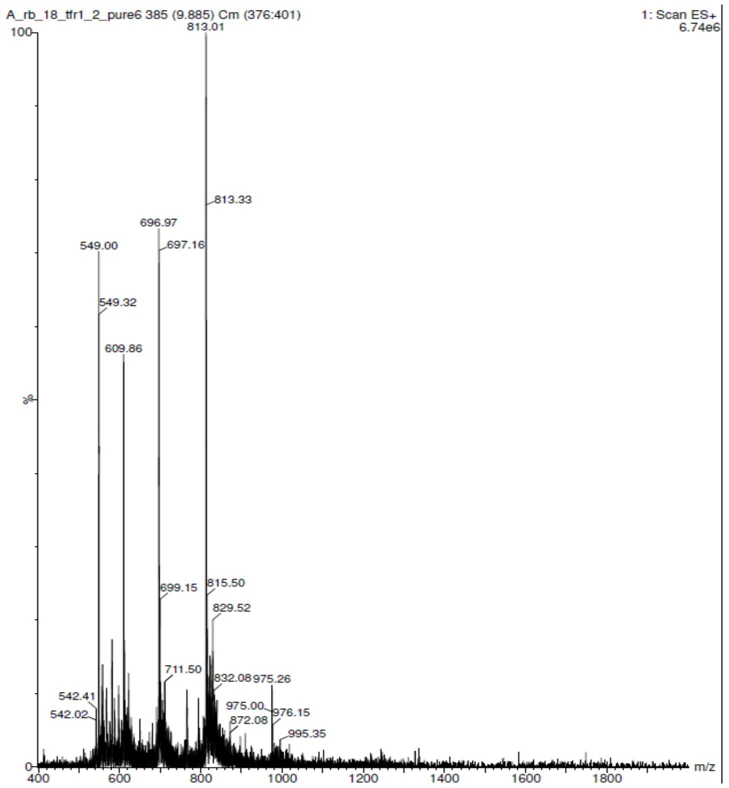

*PPII(L3X;L15K)TfRL2(K)APep2(X)*

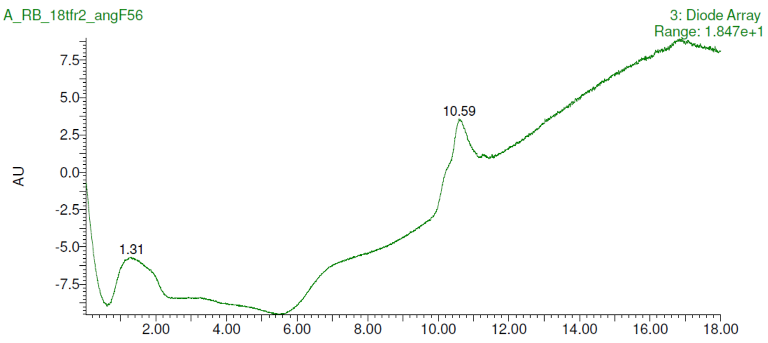


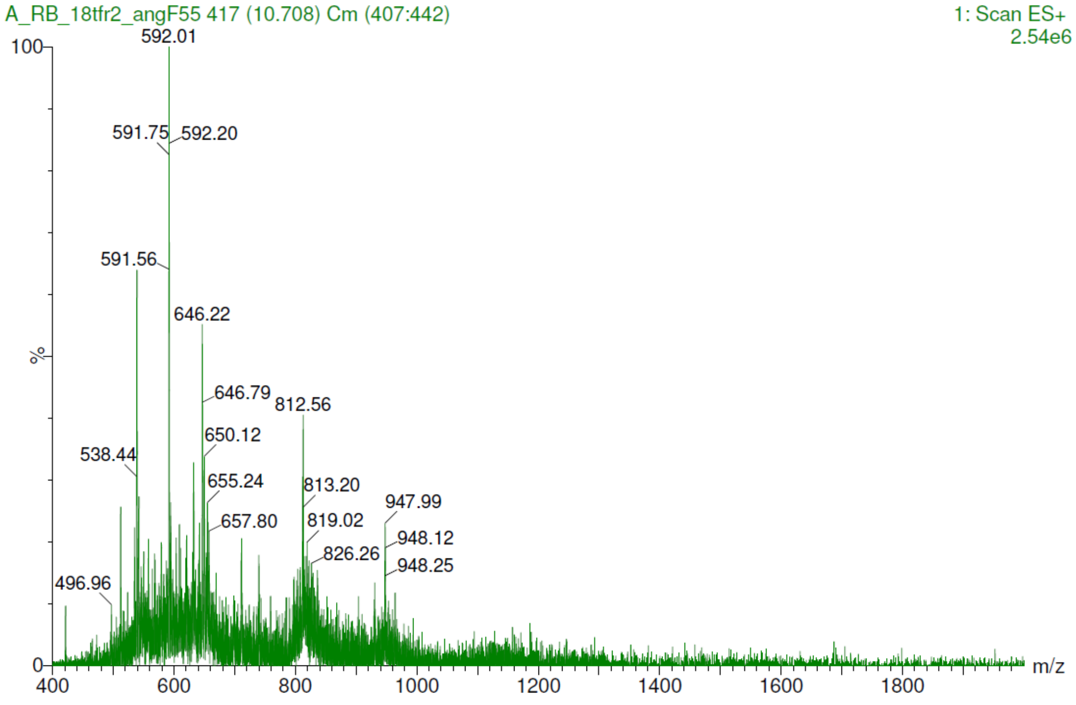

*PPII(L3X;V13K)TfRL2(K)TfRL1(X)*

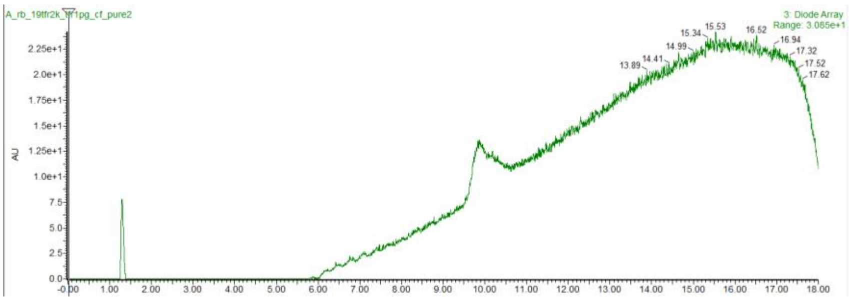


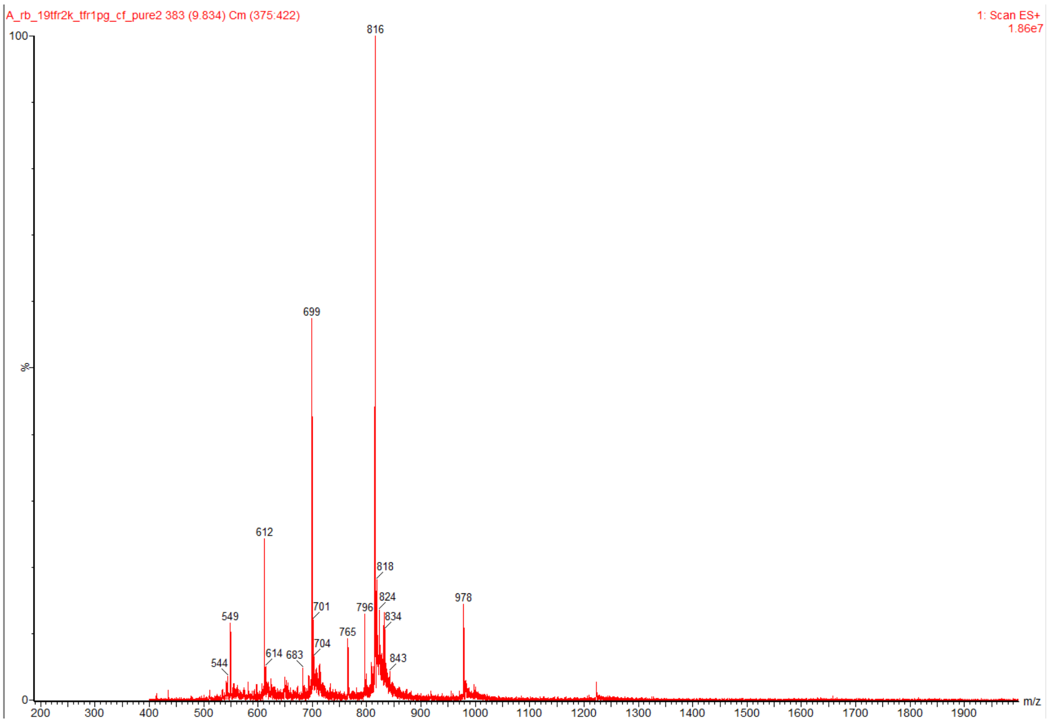

*PPII(L3X;V13K)TfRL2(K)Apep2(X)*

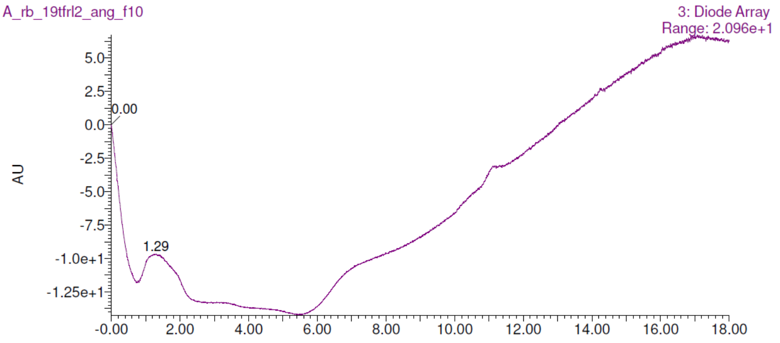


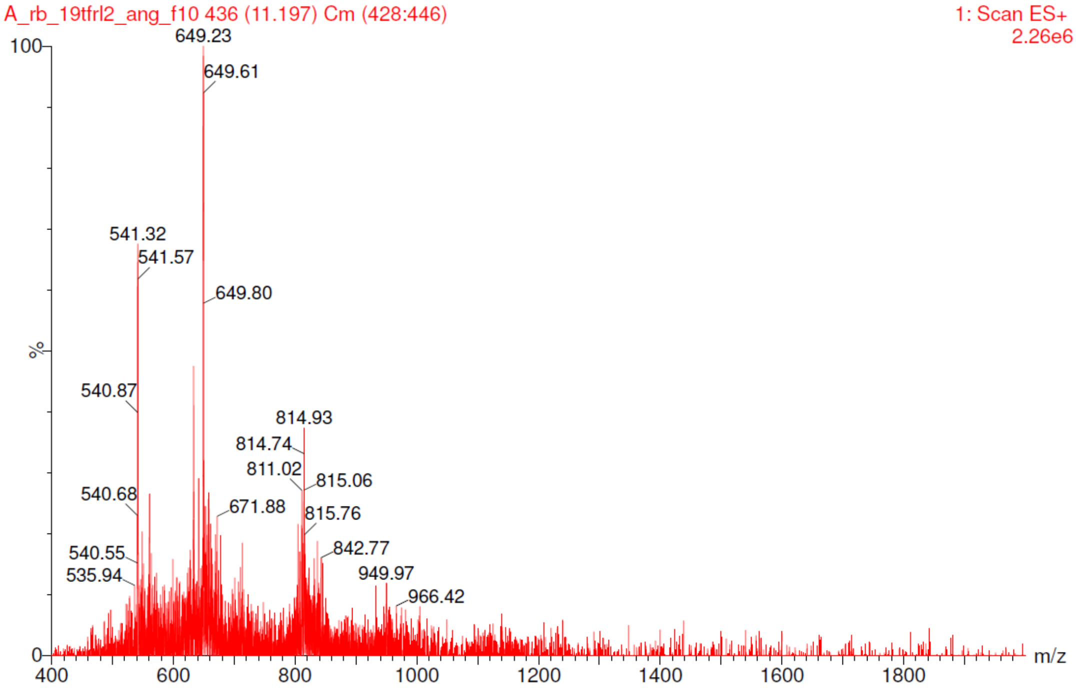

*PPII(L3X;V13K)TfRL1(X)APep2(Kx)*

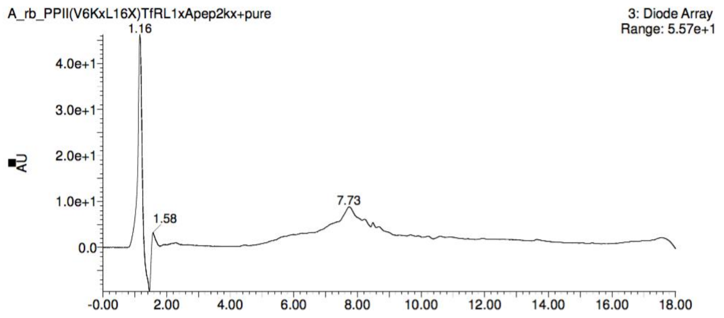


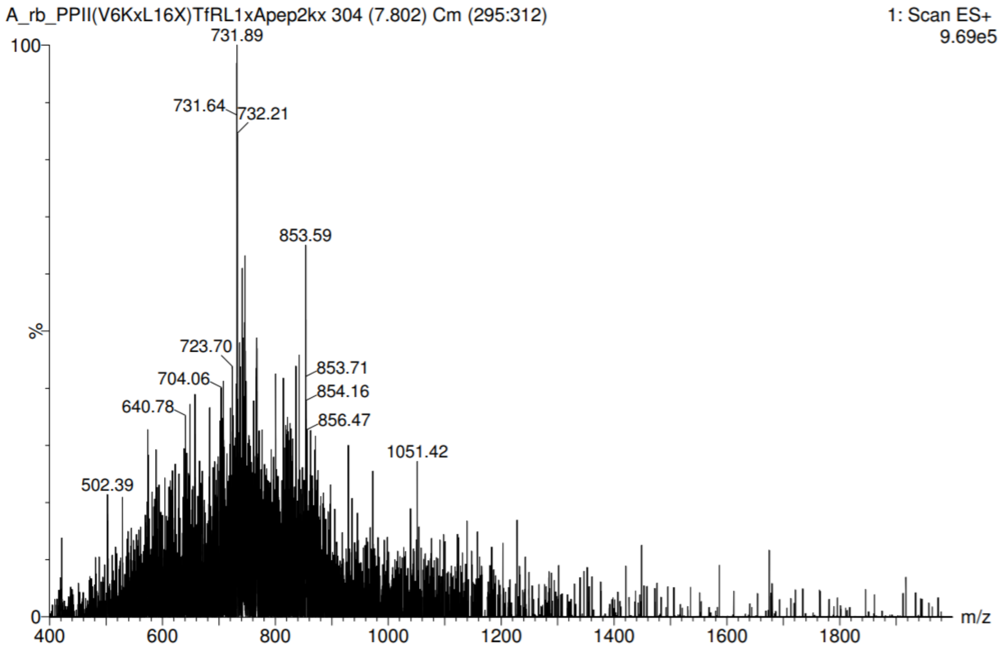

*PPII(L3X;L15K)TfRL1(X)APep2(Kx)*

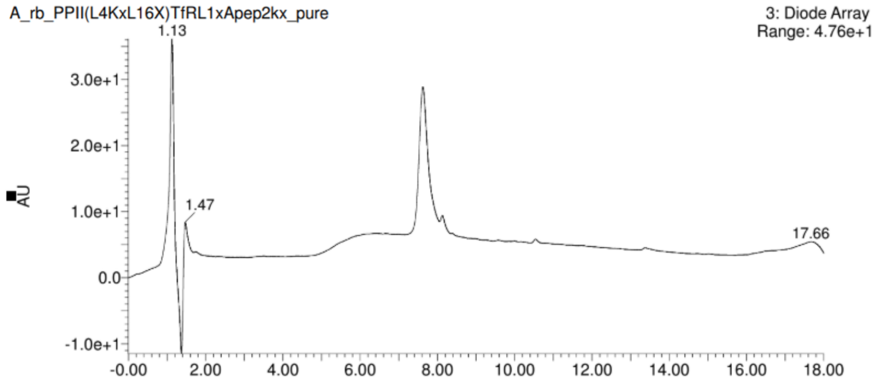


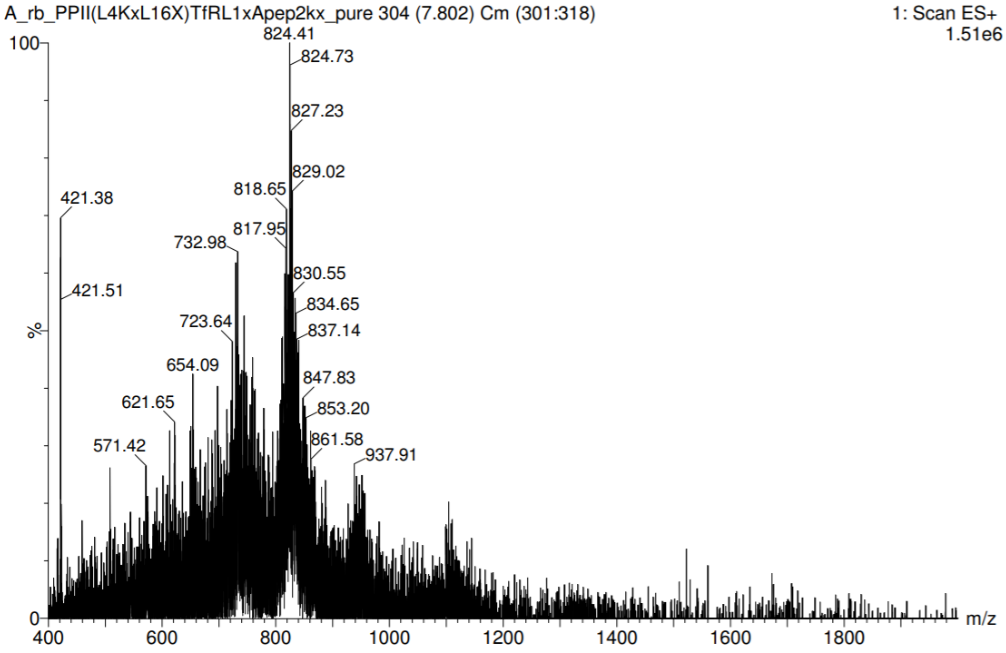


## References


1 R. C. Brown, A. P. Morris and R. G. O'Neil, *Brain research*, 2007, **1130,** 17-30

2 R. Cecchelli, S. Aday, E. Sevin, C. Almeida, M. Culot, L. Dehouck, C. Coisne, B. Engelhardt, M.-P. Dehouck and L. Ferreira, *PLoS ONE*, 2014, **9,** e99733

3 A. Patabendige, R. A. Skinner and N. J. Abbott, *Brain Research*, 2013, **1521,** 1-15.

4 J. Schindelin, I. Arganda-Carreras, E. Frise, V. Kaynig, M. Longair, T. Pietzsch, S. Preibisch, C. Rueden, S. Saalfeld, B. Schmid, J.Y. Tinevez, D.J. White, V. Hartenstein, K. Eliceiri, P. Tomancak and A. Cardona, *Nat Methods*, 2012, **28,** 676-682.

5 F. de Chaumont, S. Dallongeville, N. Chenouard, N. Hervé, S. Pop, T. Provoost, V. Meas-Yedid, P. Pankajakshan, T. Lecomte, Y. Le Montagner, T. Lagache, A. Dufour, and J.C. Olivo-Marin, *Nat Methods*, 2012, **28,** 690-696.
